# Humoral Responses Against Variants of Concern by COVID-19 mRNA Vaccines in Immunocompromised Patients

**DOI:** 10.1001/jamaoncol.2022.0446

**Published:** 2022-03-10

**Authors:** Michel Obeid, Madeleine Suffiotti, Celine Pellaton, Hasna Bouchaab, Anne Cairoli, Vanja Salvadé, Caroline Stevenel, Rosemary Hottinger, Catherine Pythoud, Lucie Coutechier, Laura Molinari, Didier Trono, Camillo Ribi, Raphael Gottardo, Craig Fenwick, Manuel Pascual, Michel A. Duchosal, Solange Peters, Giuseppe Pantaleo

**Affiliations:** 1 Service of Immunology and Allergy, Departments of Medicine and Laboratory Medicine and Pathology, Lausanne University Hospital, University of Lausanne, Lausanne, Switzerland; 2 Service of Medical Oncology, Department of Oncology, Lausanne University Hospital, University of Lausanne, Lausanne, Switzerland; 3 Service and Central Laboratory of Hematology, Departments of Oncology and Laboratory Medicine and Pathology, Lausanne University Hospital, University of Lausanne, Lausanne, Switzerland; 4 Service of Transplantation, Departments of Medicine and Surgery, Lausanne University Hospital, University of Lausanne, Lausanne, Switzerland; 5 Laboratory of Virology and Genetics, EPFL, Lausanne, Switzerland; 6 Service of Data Science and Bioinformatics, Lausanne University Hospital, University of Lausanne, Lausanne, Switzerland; 7 Swiss Vaccine Research Institute, Lausanne University Hospital, University of Lausanne, Lausanne, Switzerland

## Abstract

**Importance:**

There are limited comparative data on the durability of neutralizing antibody (nAb) responses elicited by messenger RNA (mRNA) vaccines against the SARS-CoV-2 variants of concern (VOCs) in immunocompromised patients and healthy controls.

**Objective:**

To assess the humoral responses after vaccination with BNT162b2 (Pfizer-BioNTech) or mRNA-1273 (Moderna) vaccines.

**Design, Setting, and Participants:**

In this prospective, longitudinal monocentric comparative effectiveness study conducted at the Lausanne University Hospital, binding IgG anti-spike antibody and nAb levels were measured at 1 week, 1 month, 3 months, and 6 months after vaccination with mRNA-1273 (24.6% of participants) or BNT162b2 (75.3% of participants).

**Interventions:**

All participants received 2 doses of either mRNA-1273 or BNT162b2 vaccines 4 to 6 weeks apart.

**Main Outcomes and Measures:**

The primary outcome of the study was the persistence of nAb responses against the original, nonvariant SARS-CoV-2 (2019-nCoV) and different VOCs at 6 months after vaccination. Key secondary outcomes were associations of the type of mRNA vaccine, the underlying disease, and the treatment with the response to vaccination.

**Results:**

Among the 841 participants enrolled between January 14 and August 8, 2021, the patient population comprised 637 participants (mean [SD] age, 61.8 [13.7] years; 386 [60.6%] female), and the healthy control population comprised 204 participants (mean [SD] age, 45.9 [12.0] years; 144 [70.6%] female). There were 399 patients with solid cancers, 101 with hematologic cancers, 38 with solid organ transplants, 99 with autoimmune diseases, and 204 healthy controls. More than 15 000 nAb determinations were performed against the original, nonvariant 2019-nCoV and the Alpha, Beta, Gamma, and Delta variants. The proportions of nAbs and their titers decreased in all study groups at 6 months after vaccination, with the greatest decreases for the Beta and Delta variants. For Beta, the proportion decreased to a median (SE) of 39.2% (5.5%) in those with hematologic cancers, 44.8% (2.7%) in those with solid cancers, 23.1% (8.3%) in those with solid organ transplants, and 22.7% (4.8%) in those with autoimmune diseases compared with 52.1% (4.2%) in healthy controls. For Delta, the proportions decreased to 41.8% (5.6%) in participants with hematologic cancer, 51.9% (2.7%) in those with solid cancers, 26.9% (8.7%) in those with solid organ transplants, and 30.7% (5.3%) in those with autoimmune diseases compared with 56.9% (4.1%) healthy controls. Neutralizing antibody titers decreased 3.5- to 5-fold between month 1 and month 6, and the estimated duration of response was greater and more durable among those participants vaccinated with mRNA-1273. In participants with solid cancers, the estimated duration of nAbs against the Beta variant was 221 days with mRNA-1273 and 146 days with BNT162b2, and against the Delta variant, it was 226 days with mRNA-1273 and 161 with BNT162b2. The estimated duration of nAbs in participants with hematologic cancers was 113 and 127 days against Beta and Delta variants, respectively.

**Conclusions and Relevance:**

This comparative effectiveness study suggests that approximately half of patients with hematologic cancers and solid cancers, about 70% of patients with solid organ transplants or autoimmune diseases, and 40% of healthy controls have lost nAbs against the circulating VOCs at 6 months after vaccination. These findings may be helpful for developing the best boosting vaccination schedule especially in immunocompromised patients.

## Key Points

**Question:**

Are there differences in the magnitude and durability of neutralizing antibody (nAb) responses against SARS-CoV-2 variants of concern (VOCs) according to the mRNA-1273 (Moderna) and BNT162b2 (Pfizer-BioNTech) vaccines?

**Findings:**

In this comparative effectiveness study of 637 immunocompromised patients and 204 healthy control participants who received 2 doses of messenger RNA COVID-19 vaccines, nAb responses against the Beta and Delta variants were short lived (3 to 7 months) compared with original, nonvariant SARS-CoV-2 and other variants. Higher nAb titers and longer durability of humoral responses were associated with vaccination with the mRNA-1273 vaccine.

**Meaning:**

The faster disappearance of the nAb responses in certain groups of immunocompromised patients suggests that boosting vaccine strategies need to be personalized to the underlying disease.

## Teaser

This comparative effectiveness study of immunocompromised patients and healthy controls in Switzerland examines differences in the magnitude and durability of neutralizing antibody responses against the original SARS-CoV-2 and several variants of concern according to the mRNA-1273 and BNT162b2 vaccines.

## Introduction

Immunocompromised patients with solid tumors, hematologic cancers, or autoimmune diseases and recipients of solid organ transplants are at higher risk of developing SARS-CoV-2–associated complications and higher risk of death.^1,2,3,4,5,6^ Immune responses after receipt of SARS-CoV-2 messenger RNA (mRNA) vaccines^7,8^ have been found to be decreased in these groups of patients because of the use of immunosuppressive agents such as methotrexate,^9^ antimetabolites,^2,10^ Bruton tyrosine kinase inhibitors,^11^ Bcl-2 antagonists,^11^ or anti-CD20 therapy.^12,13,14^ Recent studies indicate that SARS-CoV-2 variants of concern (VOCs) may decrease vaccine-induced protective immunity,^15^ increase transmissibility,^16,17^ lead to resistance to human monoclonal antibodies, and decrease sensitivity to convalescent plasma and sera among vaccinated individuals.^18,19,20,21,22,23,24^ Furthermore, the VOCs increase the risk of breakthrough infections among health care workers.^25^ In a study by Abu-Raddad et al, the incidence of breakthrough infections appeared to accelerate with time after the second dose of vaccine among those with no prior infection, suggesting a waning of vaccine-induced immunity over time.^26^

Waning of binding IgG anti-spike (anti-S) antibody levels and neutralizing titers occurs at 3 and 6 months after vaccination among healthy controls^15,27,28,29^ and health care workers.^30^ A study by Eliakim-Raz et al found that binding IgG anti-S titers were significantly lower in patients with cancer compared with those in healthy controls, suggesting a faster decrease of the humoral response in these patients.^31^ Among the VOCs, the Beta variant had the lowest response to the BNT162b2 vaccine (Pfizer-BioNTech) and a higher resistance to convalescent or vaccine sera.^22,32,33^ These studies have raised concern for immunocompromised individuals, and limited data are available on the durability of neutralizing antibody (nAb) responses against the VOCs in these patients.

In this study (the ImmunoVax study), we sought to characterize the humoral response, as measuring both binding IgG antibodies and nAbs, against the original, nonvariant SARS-CoV-2 (2019-nCoV) and the Alpha, Beta, Gamma, and Delta variants and the durability of the humoral response at 6 months after vaccination.

## Methods

### Study Design and Population

Between January 14 and August 8, 2021, participants were enrolled in the ImmunoVax study, a single-center, prospective, longitudinal comparative effectiveness study of immunocompromised patients with solid cancers, hematologic cancers, autoimmune diseases, or solid organ transplants and healthy controls who received 2 doses of mRNA COVID-19 vaccines. All participants gave written informed consent. All participants with positive serological test results indicative of past SARS-CoV-2 infection at baseline were excluded from the immunologic analyses. Laboratory personnel were blinded to the origin of samples (study group and time of the collection). The study was approved by the institutional review board of the Lausanne University Hospital and is registered with the local ethics committee. This study follows the International Society for Pharmacoeconomics and Outcomes Research (ISPOR) reporting guideline.^34^

### Serologic Assays and Procedures

Participants received 2 doses of BNT162b2 or mRNA-1273 (Moderna) administered intramuscularly 19 to 131 days apart (mean [SD] 31.8 [7.0] days) or a single dose if participants had had a previous SARS-CoV-2 infection (12 participants). Binding IgG anti-S and anti-nucleocapsid antibody and nAb levels were determined using 2 Luminex (Luminex Corp)-based assays recently developed in our laboratory.^35,36^

### Outcomes

The co-primary outcomes were seroconversion, as shown by the detection of binding IgG anti-S antibodies, and nAb responses against the VOCs in the study groups after vaccination with BNT162b2 or mRNA-1273 vaccine. The secondary outcome was safety after each vaccine dose, measured according to adverse events.

### Statistical Analysis

Spike–angiotensin-converting enzyme 2 half maximal inhibitory concentration (IC_50_) dilution values and binding IgG anti-S antibody ratios were log_10_ transformed for visualization and statistical modeling. Differences in anti-S nAbs IC_50_ dilution values and binding IgG anti-S antibody ratios between groups (ie, study groups or vaccine type) were tested using linear regression models for individual time points (at 1 month and 3 months after the second dose of vaccine) and adjusting for age (<60 or ≥60 years) and sex. When comparing responses across time points, a mixed effects model with a subject random effect was used instead. Resulting *P* values were adjusted for multiple testing using the Benjamini-Hochberg false discovery rate. Tests were 2-tailed, with an adjusted *P* <  .05 considered statistically significant. The proportions of individuals with nAbs (anti-S nAbs IC_50_ dilution values ≥0) were calculated by taking into account patients scored as IgG negative (IgG anti-S antibody ratio values <5.19 U/mL) for which no neutralization tests were performed. Error bars for proportions were calculated as [*p*(1 − *p*)/*n*]^1/2^, where *p* is the proportion and *n* is the number of samples.

Summary statistics for binding IgG and neutralizing antibodies, such as median and 95% CIs for the median were calculated on nontransformed data. The binding IgG and neutralizing antibody durations (in days after second dose) were estimated with linear regression models using time as continuous covariate: y = b0 + b1 time.

For each response variable (binding IgG and neutralizing antibodies), we estimated the intercept (b0 coefficient) and the slope (b1). The model coefficients were used to estimate the time at which the level of response variable was equal to the cutoff of positivity; that is, 50 for nAbs and 5.19 for binding IgG antibodies: time = (y − b0)/b1.

To determine the 80% CIs for the estimated time at which the antibody level data fall below the lower limit, a bootstrapping approach was used to randomly resample 10 000 times (with replacement) the neutralization titers or IgG ratios. These randomly generated samples from the original data were used to fit the model and estimate the 10% and 90% quantiles used to define an 80% CI. We decided to use an 80% CI to improve visibility (eTable 5 in the Supplement). Otherwise, the intervals could be quite wide, extending the right range of the figure where no data are observed. These CIs are simply used to summarize the uncertainty associated with each point estimate and are not use for statistical inference. The differences in the number of safety events per vaccine type were evaluated using the Fisher exact test. Statistical analysis was performed using R software, version 4.1.1 (R Foundation for Statistical Computing). Further details of the statistical analysis are found in eMethods in the Supplement.

## Results

A total of 887 participants were enrolled in this prospective longitudinal comparative effectiveness study. After exclusions (43 patients withdrew from the study, and 3 could not be analyzed), the patient population comprised 637 participants (mean [SD] age, 61.8 [13.7] years [range, 19.4-92.5 years]; 386 [60.6%] female and 251 [39.4%] male; The healthy control population comprised 204 participants (mean [SD] age, 45.9 [12.0] years [range, 24.4-85.5 years]; 144 [70.6%] female and 60 [29.4%] male) (eFigure 1 in the Supplement). Information on the different treatments is provided in eTable 2 in the Supplement. Among the 637 patients, 399 patients (62.6%) were diagnosed with solid cancers, 101 patients (15.9%) had hematologic cancers, 99 patients (15.5%) had autoimmune diseases, 38 patients (6.0%) received solid organ transplants. Three hundred and ninety-one patients (61.4%) were undergoing active systemic treatment at the time of vaccination: 200 patients (31.4%) with solid cancers, 57 (8.9%) with hematologic cancer, 96 (15.1%) with autoimmune diseases, and 38 (6.0%) with solid organ transplants. The pathological conditions and treatments are detailed in eTables 1 and 2 in the Supplement.

Blood samples were collected at baseline before the first vaccine dose (visit 1) and at 1 week (visit 2), 1 month (visit 3), 3 months (visit 4), and 6 months (visit 5) after vaccination. Among the 841 active participants, 54 participants (12 healthy controls, 6 with solid organ transplants, 7 with hematologic cancer, 7 with autoimmune diseases, and 22 with solid cancers) having a positive serologic test result for binding IgG antibodies at visit 1, indicative of prior and/or ongoing SARS-CoV-2 infection, remained in the study but were excluded from the immunologic analyses. A total of 631 patients (75.3%) received BNT162b2, and 207 (24.6%) received mRNA-1273; information for 3 participants was unknown (eTable 1 in the Supplement). Five participants (0.6%: 2 healthy controls, 2 with autoimmune diseases, and 1 with solid cancer) were diagnosed with SARS-CoV-2 infection during the study. The interim results of the humoral response for participants up to visit 5 are reported in the present study, including data obtained up to December 18, 2021. At the time of analysis, 772 participants were included for immunologic analyses, and 748 participants had completed visit 2, 751 had completed visit 3, 720 had completed visit 4, and 676 had completed visit 5.

The analyses of the antibody responses after vaccination were adjusted for age and sex. The ranges for the collection of blood after the second dose of vaccine across the study groups were 31 or 32 days at visit 3, 92 or 93 days at visit 4, and 185 to 187 at visit 5 (because there were no significant differences in the humoral response between visit 2 and visit 3, the responses at visit 3, visit 4, and visit 5 are shown). At 1 month after the second vaccine dose among 100% of the healthy controls, 100% of participants with untreated solid cancers and a median (SE) 98.3% (1.0%) of participants with treated solid cancers and 95.0% (3.40%) of participants with untreated hematologic cancer had positive diagnostic results for binding IgG anti-S antibodies (eFigure 2A in the Supplement). The median (SE) percentages were lower among the participants with solid organ transplants (65.5% [8.8%]), autoimmune diseases (81.8% [1.0%]), and treated hematologic cancers (86.0% [04.9%]). Of note, the levels of binding IgG anti-S antibodies were significantly lower in the participants with solid organ transplants (median, 81.1 U/mL; 95% CI, 1.9-527.9 U/mL), autoimmune diseases (median, 1623.9 U/mL; 95% CI, 882.1-2309.7 U/mL), and treated hematologic cancers (median, 1383.0 U/mL; 95% CI, 582.7-2224.2 U/mL) compared with the healthy controls (median, 1900.4 U/mL; 95% CI, 1816.1-2119.8) (*P* < .001 for all) (eFigure 2B in the Supplement), whereas the levels were not significantly different between the mRNA-1273 and BNT162b2 vaccines (eFigure 2C in the Supplement).

**Figure 1.**
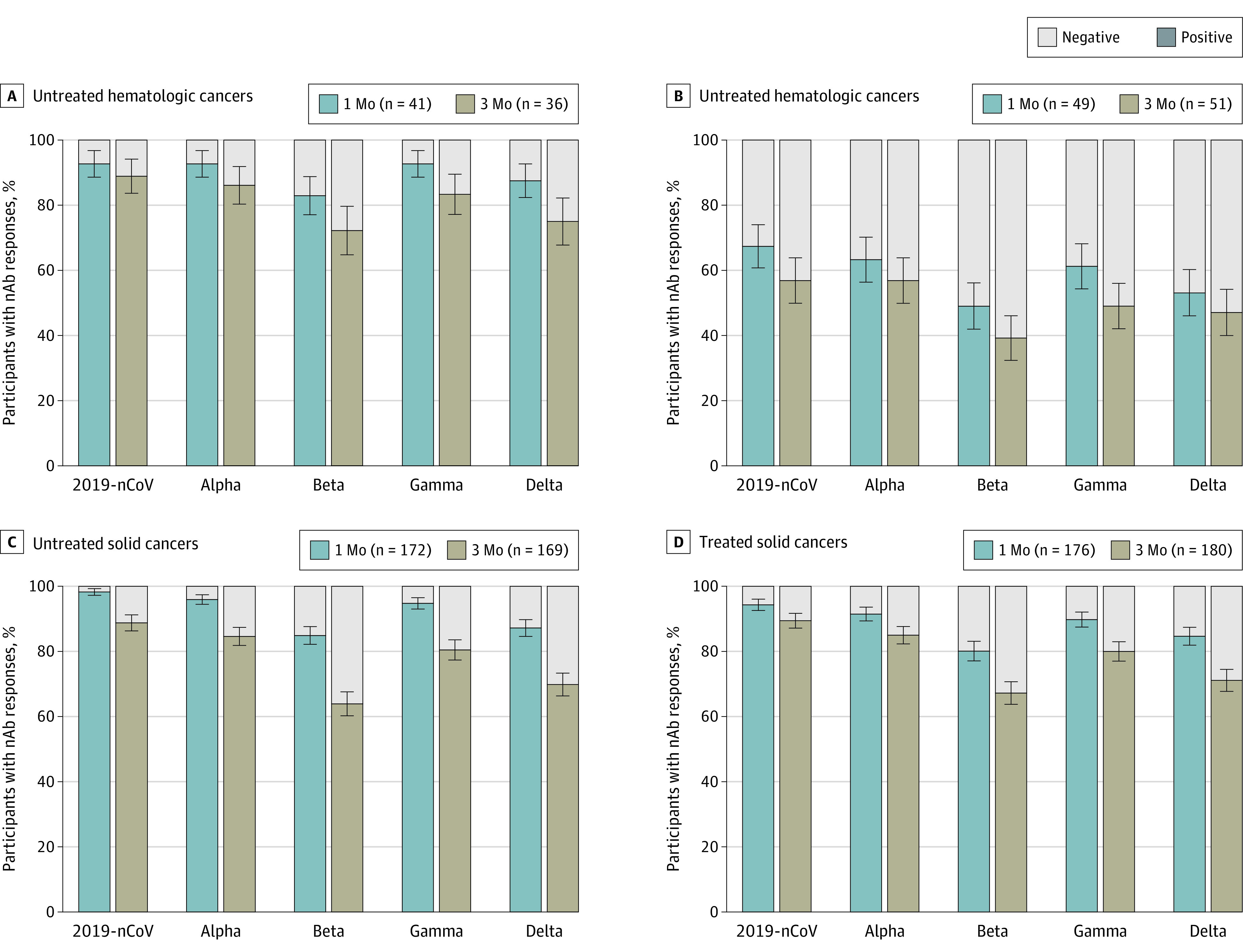
Percentages of Participants With Neutralizing Antibody (nAb) Responses at 1 Month and 3 Months After the Second Vaccine Dose Neutralizing antibody responses were measured against 2019-nCoV (the original, nonvariant SARS-CoV-2) and the different variants of concern. Data are expressed as IC_50_ (half maximal inhibitory concentration) dilutions. Negative (gray bars) indicates IC_50_ titers <50 dilutions; positive (colored bars) indicates IC_50_ titers >50 dilutions. Values are median (SE, denoted by whiskers).

We next determined the nAb responses to vaccination against 2019-nCoV and the Alpha, Beta, Gamma, and Delta variants using a cell- and virus-free assay recently developed in our laboratory^35^ cross-validated with the criterion standard live virus assay. Almost the totality of healthy controls had nAbs against 2019-nCoV and the different variants (ranging from a median [SE] of 95.7% [1.49%] to 100% [0%]) (eFigure 3 in the Supplement) at 1 month after vaccination. The median (SE) proportions of responders at 1 month after vaccination were slightly lower in participants with untreated (range, 84.9% [2.73%]-98.3% [0.99%]) and treated solid cancers (range, 80.1% [3.01%] to 94.3% [1.75%]), lower in those with untreated hematologic cancer (range, 82.9% [5.88%] to 92.7% [4.06%]), and still lower in participants with treated hematologic cancers (range, 49.0% [7.14%] to 67.3% [6.70%]), participants with autoimmune diseases (range, 48.2% [5.42%] to 70.6% [4.94%]), and participants with solid organ transplants (range, 17.2% [7.01%] to 37.9% [9.01%]) (Figure 1; eFigure 3 in the Supplement). At 3 months after vaccination, the percentages of individuals with nAb responses against the Beta and Delta variants decreased in all study groups and decreased substantially in the treated groups: median (SE), 39.2% (6.84%) for Beta and 47.1% (6.99%) for Delta in participants with treated hematologic cancers, 67.2% (3.50%) for Beta and 71.1% (3.38%) for Delta in participants with treated solid cancers, 32.5 (5.24%) for Beta and 38.8% (5.45%) for Delta in participants with autoimmune diseases, and 9.7% (5.32%) for Beta and 16.1% (6.60%) for Delta in participants with solid organ transplants (Figure 1; eFigure 3 in the Supplement).

We subsequently evaluated the magnitude of the nAb responses as measured by IC_50_ dilutions greater than 50, the cutoff for a positive diagnostic test result. At 1 month and 3 months after vaccination, the IC_50_ titers against 2019-nCoV were significantly lower in participants with solid organ transplants, autoimmune diseases, treated hematologic cancer, and untreated solid cancers compared with the other groups.

For example. at 1 month after vaccination, the IC_50_ titers against 2019-nCoV were significantly lower in participants with solid organ transplants (median, 16.5; 95% CI, 8.5-68.1; *P* < .001), autoimmune diseases (median, 208.3; 95% CI, 164.4-373.5; *P* = .02), treated hematologic cancers (median, 255.4; 95% CI, 136.2-431.3; *P* = .02), and untreated solid cancers (median, 465.1; 95% CI, 406.4-529.3; *P* = .02) compared with healthy controls (median, 531.9; 95% CI, 483.1-584.4), untreated hematologic cancers (median, 490.4; 95% CI, 290.5-707.3), and treated solid cancers (median, 475.9; 95% CI, 401.2-551.2).

Similarly,the IC_50_ titers against the Delta variant were significantly lower in participants with solid organ transplants (median, 10.2; 95% CI, 3.5-16.5; *P* < .001), autoimmune diseases (median, 64.4; 95% CI, 36.4-80.5; *P* < .001), treated hematologic cancers (median, 77.1; 95% CI, 36.1-143.3; *P* < .001) compared with healthy controls (median 197.1; 95% CI, 183.2-216.4), untreated solid cancers (median, 163.5; 95% CI, 142.4-185.1), treated solid cancers (median, 172.3; 95 CI, 134.3-188.5), and untreated hematologic cancers (median, 178.5; 95% CI, 129.2-253.1) (eTable 3 in the Supplement).

**Figure 2.**
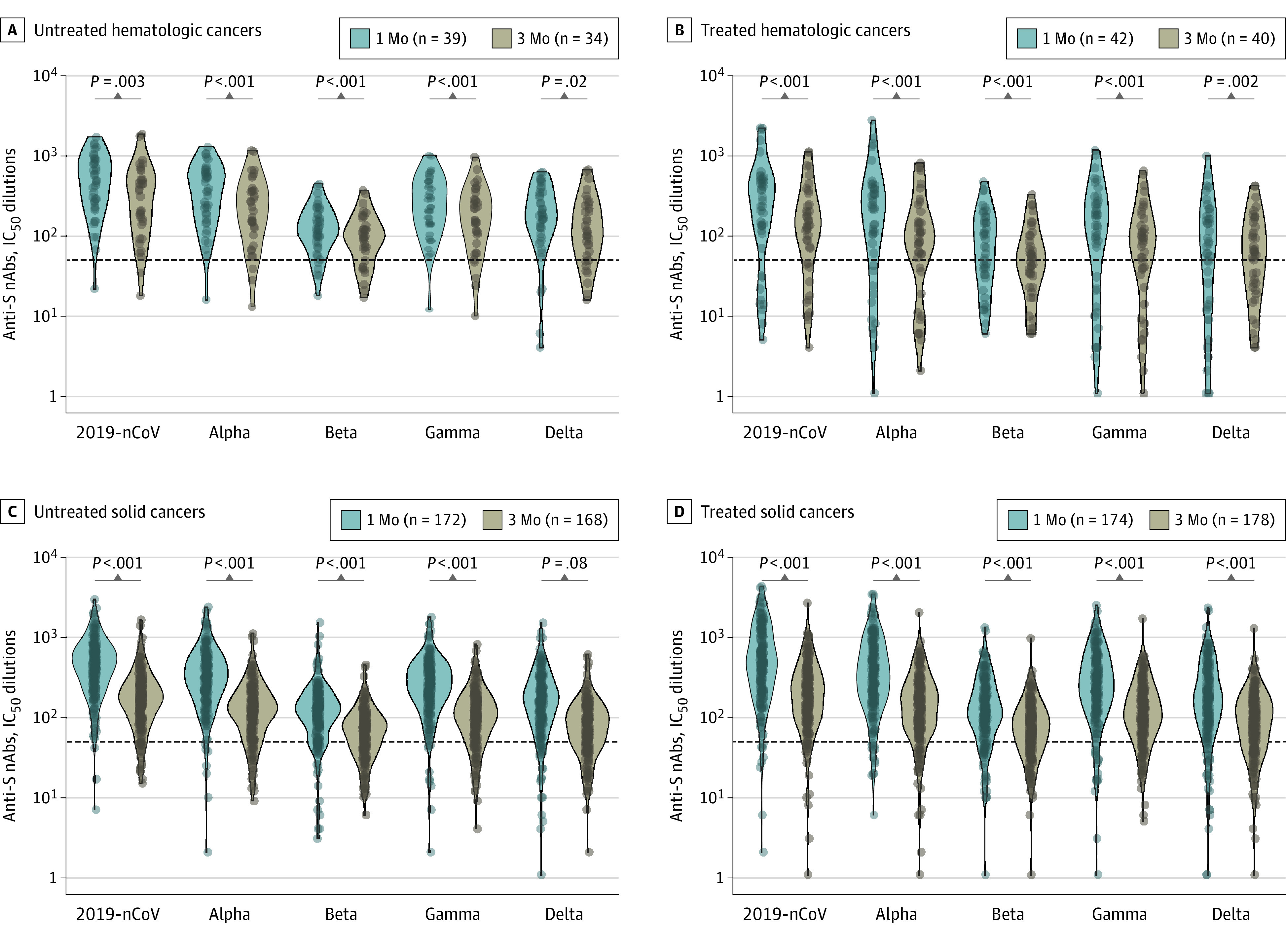
Levels of Neutralizing Antibody (nAb) Responses at 1 Month and 3 Months After the Second Vaccine Dose Neutralizing antibody responses are measured by half maximal inhibitory concentration (IC_50_) dilutions. The dotted lines indicate the threshold positivity of the assay (ie, IC_50_ greater than 50 dilutions); IC_50_ dilutions were log_10_ transformed for analysis. Resulting *P* values were adjusted for multiple testing using the Benjamini-Hochberg false discovery rate. Anti-S indicates IgG anti-spike; 2019-nCoV, the original, nonvariant SARS-CoV-2.

The IC_50_ titers against the Beta and Delta variants were about 3- to 4-fold lower compared with those against 2019-nCoV in all groups, with significant decreases (1.7- to 2.5-fold) in all the VOCs titers observed between month 1 and month 3 in all patient groups (with the exception of participants with solid organ transplants) (Figure 2; eFigure 4 in the Supplement). These responses were differentially associated with B-cell–depleting therapies and other classes of potent immunosuppressive agents, and there was a trend toward better humoral responses in participants younger than 65 years and in female participants (eTable 4 in the Supplement).

**Figure 3.**
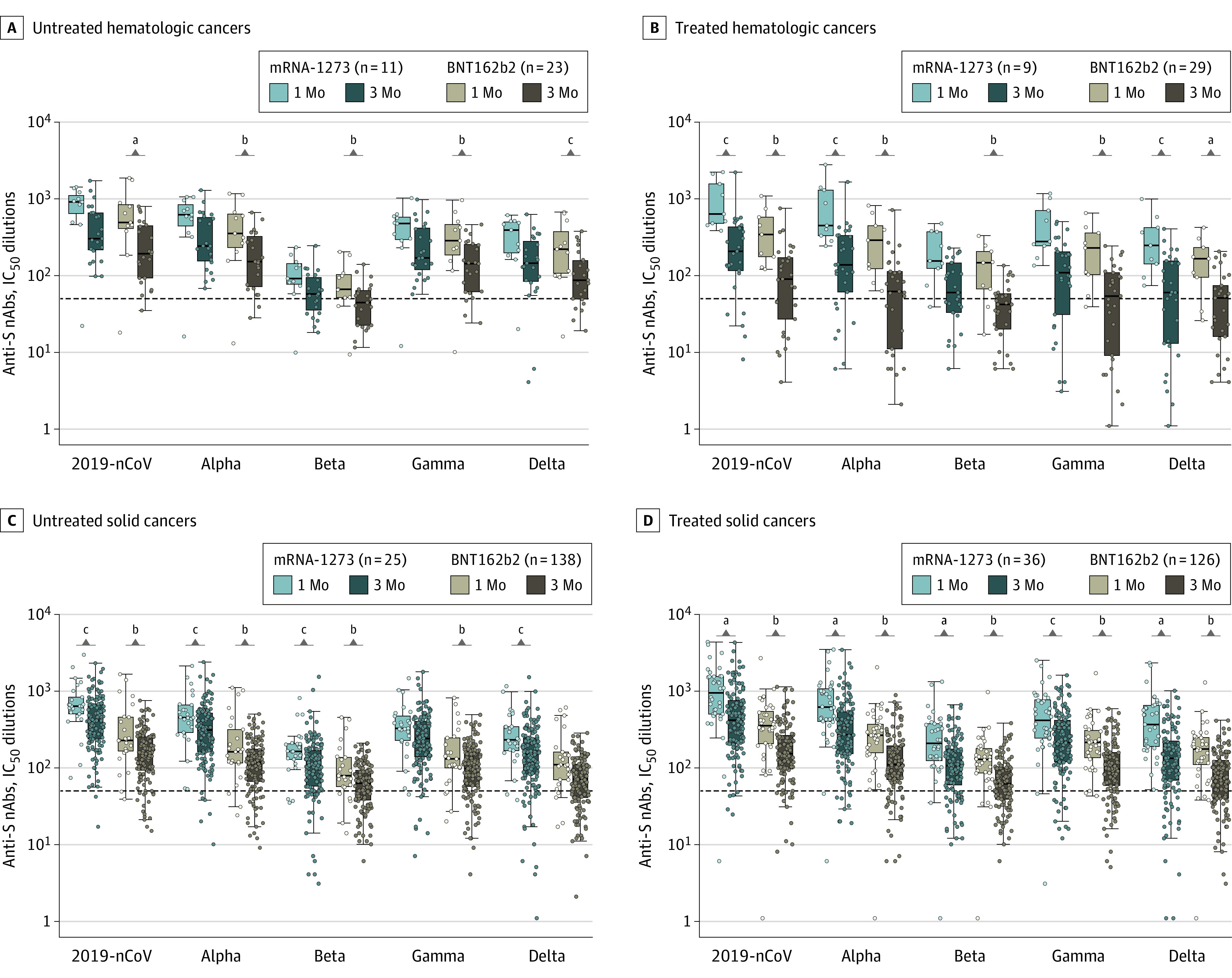
Levels of Neutralizing Antibody (nAb) Responses After the Second Dose of the mRNA-1273 or BNT162b2 Vaccine Neutralizing antibody responses are measured by half maximal inhibitory concentration (IC_50_) dilutions. The dotted lines indicate the threshold positivity of the assay (ie, IC_50_ >50 dilutions); IC_50_ dilutions were log_10_ transformed for analysis. Resulting *P* values were adjusted for multiple testing using the Benjamini-Hochberg false discovery rate. Values are median (SE, denoted by whiskers). Additional information on *P *values is provided in the eLegend for eFigure 3 in the Supplement. Anti-S indicates IgG anti-spike; 2019-nCoV, the original, nonvariant SARS-CoV-2. ^a^*P *< .01. ^b^*P* < .001. ^c^*P *< .05.

The IC_50_ titers against 2019-nCoV and the VOCs were consistently higher (3.0- 4.0-fold) in the participants vaccinated with the mRNA-1273 vs BNT162b2 in all study groups at 1 month and 3 months after vaccination (Figure 3; eFigure 5 in the Supplement). To appreciate further the differences between the 2 mRNA vaccines, nAb responses were stratified on the basis of different IC_50_ titers. Of note, the percentage of individuals with IC_50_ titers lower than 50 (negative response) was higher in those vaccinated with BNT162b2 (eFigure 6 in the Supplement).

**Figure 4.**
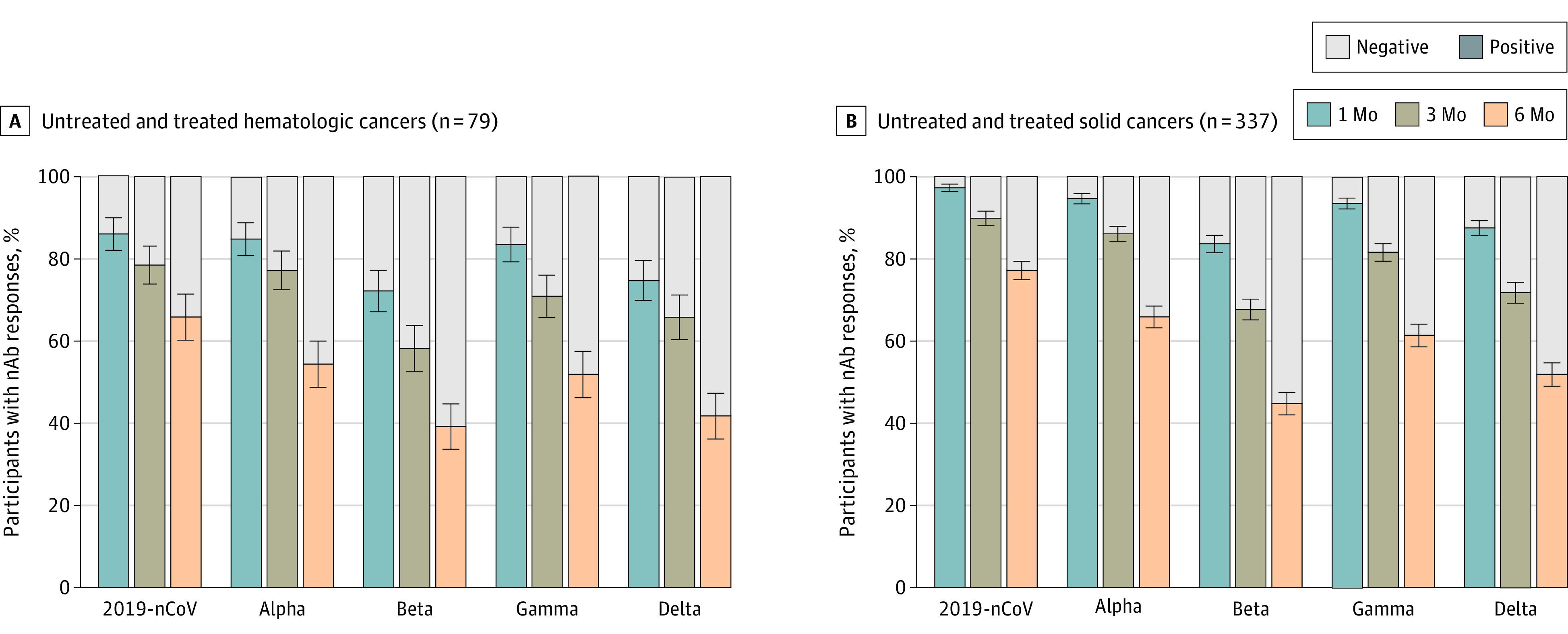
Percentages of Participants With Neutralizing Antibody (nAb) Responses at 1 Month, 3 Months, and 6 Months After the Second Vaccine Dose Untreated and treated hematologic cancers and solid cancers participants were combined for the analysis within each group. Negative (gray bars) indicates IC_50_ titers <50 dilutions; positive (colored bars) indicates IC_50_ titers >50 dilutions. Values are median (SE, denoted by whiskers). 2019-nCoV indicates the original, nonvariant SARS-CoV-2.

A fraction of participants (n = 661) with matched samples at 1 month, 3 months, and 6 months were analyzed for nAbs at 6 months after vaccination. The percentage of individuals with IC_50_ titers lower than 50 decreased substantially against the Beta and Delta variants (range, 22%-30%) at 6 months in the participants with solid organ transplants and autoimmune diseases (eFigure 7 in the Supplement). The decrease was more contained in the groups with hematologic cancers (median [SE], 39.2 [5.5%] for Beta and 41.8 [5.6%] for Delta), and solid cancers (44.8 [2.7%] for Beta and 51.9% [2.7%] for Delta) (Figure 4) and in the healthy controls (52.1 [4.2%] for Beta and 56.9 [4.1%] for Delta) (eFigure 7 in the Supplement).

We then determined the time to negative diagnostic levels of binding IgG anti-S antibodies (<5.19 U/mL) and nAbs (IC_50_ titers <50). A linear regression model using time as continuous covariate (number of days after vaccination) was used for generating estimates. Among the different groups with matched data available at 1 month, 3 months, and 6 months after vaccination, 278 participants with solid cancers, 49 with hematologic cancers, and 101 healthy controls received BNT162b2, whereas 78 participants with solid cancers, 30 with hematologic cancers, and 43 healthy controls received mRNA-1273. Separate analysis between the 2 vaccines within the each group was performed only in participants with solid cancers and healthy controls because of the limited number of participants in the other groups, and no analysis was possible in participants with solid organ transplants and autoimmune diseases. The time to negative diagnostic level of binding IgG anti-S antibodies was estimated to be 1055 days in participants with solid cancers vaccinated with the mRNA-1273 vaccine and 578 days in those vaccinated with the BNT162b2 vaccine (eFigure 8 and eTable 5 in the Supplement).

**Figure 5.**
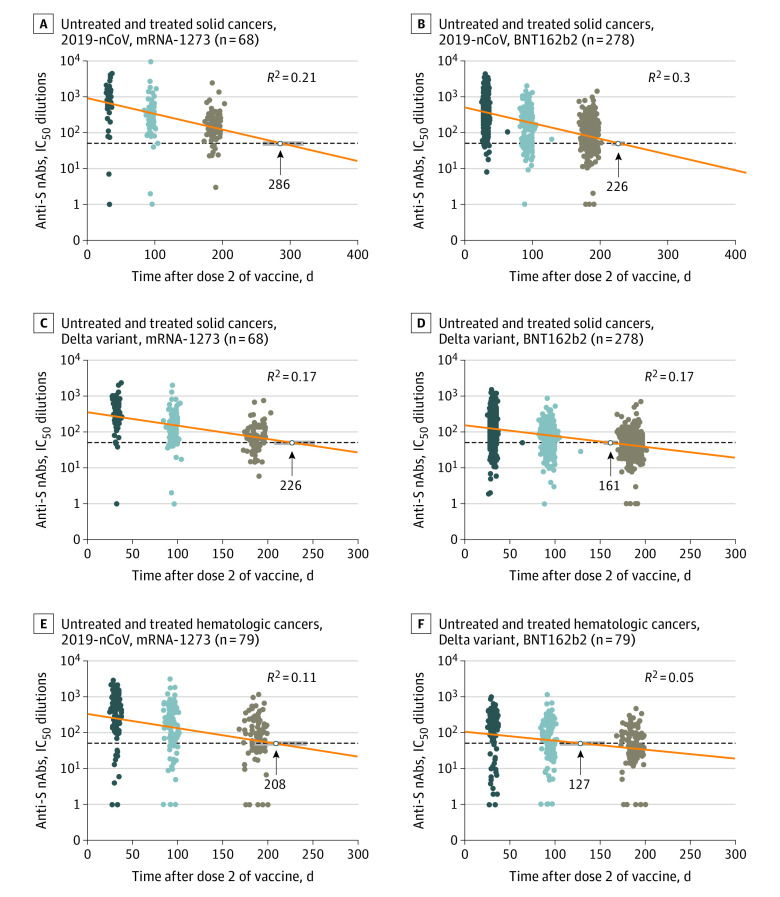
Estimates of the Duration of Neutralizing Antibody (nAb) Responses for Both Vaccines at 6 Months After the Second Vaccine Dose Among participants, 278 with solid cancers received BNT162b2, and 68 received mRNA-1273. Cumulative analyses of the two vaccines are shown for the 79 patients with hematologic cancers. A-D, Duration of nAb responses against the original, nonvariant SARS-CoV-2 (2019-nCoV) and the Delta variant in participants with solid cancers. E and F, Duration of nAbs responses in participants with hematologic cancers. The nAb duration in days was estimated by linear regression models using time as a continuous covariate (the number of days to 1 month, 3 months, and 6 months after vaccination). Anti-S indicates IgG anti-spike.

The estimated time to negative diagnostic level of nAbs was much shorter compared with binding IgG antibodies (Figure 5; eFigure 8 in the Supplement); the times to IC_50_ titers lower than 50 against 2019-nCoV were 286 and 226 days in participants with solid cancers vaccinated with mRNA-1273 and BNT162b2, respectively. The shortest estimated durations of response for nAbs were observed against the Beta variant (221 days with mRNA-1273 and 146 days with BNT162b2) and against the Delta variant (226 days with mRNA-1273 and 161 with BNT162b2). The estimated durations of responses for nAbs against the Alpha and Gamma variants were slightly shorter than against 2019-nCoV (Figure 5A; eFigure 8 in the Supplement). Overall, the estimates of the duration of binding IgG and neutralizing antibody responses in healthy controls were similar to those in participants with solid cancers (eFigure 9 in the Supplement). The estimated duration of both binding IgG and and neutralizing antibodies was shorter in participants with hematologic cancers (Figure 5B; eFigure 10 in the Supplement), with 592 days for binding IgG anti-S antibodies, 208 days for nAbs against 2019-nC0V, and 113 and 127 days against Beta and Delta variants, respectively.

Of note, there was a 4.4- to 5.1-fold decay rate in nAbs between 1 month and 6 months for 2019-nCoV and the Alpha and Gamma variants and a 3.5-fold decay rate for the Beta and Delta variants and a 4.5- to 5.4-fold decay rate for binding IgG antibodies (eTable 3 in the Supplement). The type of vaccine and the underlying disease did not appear to have any influence on the decay rate.

Adverse event analyses are provided for 839 participants, collected at visits 2 and 3. The reactogenicity was generally mild or moderate with no severe or grade 4 symptoms reported or serious adverse events or deaths. As reported previously,^7,8^ the local reactogenicity was similar after the first and second dose, whereas systemic reactogenicity was more common and severe after the second dose. At visit 2, 82.5% of participants reported local and 67.5% reported systemic reactions after mRNA-1273 (eFigure 11A in the Supplement) vs 63.4% and 49.7%, respectively, after BNT162b2 (eFigure 11B in the Supplement). Overall, reactogenicity events were transient and resolved within a few days. Only 30 participants reported persistent reactions at visit 3, especially fatigue (11 participants), headache (7 participants), and persistent lymphadenopathy (2 participants). For local reactogenicity, more mRNA-1273 than BNT162b2 recipients reported pain (74.3% vs 59.2%), redness (14.6% vs 3.6%), and swelling (12.6% vs 2.8%) (eFigure 11A in the Supplement). Similarly, more mRNA-1273 recipients reported systemic reactions, including fatigue (36.9% vs 26.6%), fever (36.4% vs 9%), muscle pain (34.5% vs 13%), headache (28.6% vs 15.3%), joint pain (13.6% vs 7.1%), nausea (9.2% vs 4.9%), chills (6.8% vs 3.2%), and vomiting (3.4% vs 1.1%). No myocarditis or anaphylactic reactions were been reported (eFigure 11B in the Supplement).

## Discussion

In this comparative effectiveness study, we found that nAb IC_50_ titers were up to 4-fold lower against the Beta and Delta variants at 1 month after the second dose of vaccine, and we found a continuous waning of the nAbs over 6 months^15,28,29,31^ and a shorter duration of the responses against Beta and Delta variants vs 2019-nCoV.

Owing to the complexity of the pseudovirus and/or live virus neutralization assays, it has been proposed that binding IgG anti-S antibodies provide insights on the persistence of nAbs over time.^37,38^ However, in our study the estimated duration of the binding IgG anti-S antibodies was about 5- to 6-fold longer compared with nAbs depending on the type of vaccine in the different study groups. In contrast, the duration of nAbs against 2019-nCoV was estimated to be 8 to 9 months and only 3 to 6 months against the Beta and Delta variants. Therefore, binding IgG anti-S antibodies may not reflect the long-term persistence of nAbs against the VOCs.

Of note, nAb responses were of substantially greater magnitude and longer duration (7 to 9 weeks) after vaccination with the mRNA-1273 compared with the BNT162b2 vaccine in all study groups. The differences are likely associated with the higher concentration (greater than 3-fold) of mRNA-1273 compared with the BNT162b2 vaccine. Our results further support the recent findings of a study that included a small number of healthy donors (31 participants) that found greater responses with the mRNA-1273 vaccine.^37^

In contrast to immune checkpoint inhibitors, endocrine therapy, biologic disease-modifying antirheumatic drugs, or targeted therapies, treatments with anti-CD20 antibodies, Bruton tyrosine kinase inhibitors, Bcl-2 antagonists, anti-CD38 therapy, or antimetabolites have been associated with impaired response to vaccination, as shown in previous studies.^11,12,13,14,39^ Our study suggests that the immunocompromised patients mostly affected are those with solid organ transplants, followed by patients with autoimmune diseases and hematologic cancers. Vaccine-induced immune responses in patients with solid cancers were similar overall to those in healthy participants, likely owing to a better function of the immune system and the lack of treatments with B-cell–depleting therapies or other potent immunosuppressive agents.

### Strengths and Limitations

This study has several strengths. To our knowledge, it has the largest collection of immunocompromised individuals studied for response to COVID-19 vaccination and for the durability of the humoral response. It is also the first study, to our knowledge, to evaluate the vaccine-induced antibody responses against the original 2019-nCoV and the VOCs and presents the largest number of determinations of nAbs induced by vaccination (approximately 15 000). This large data set has been important for generating the estimates of the durability of the antibody responses against the different VOCs among the different groups of immunocompromised patients.

This study also has limitations. A main limitation of our study is that it is not suitable for determining the threshold titers of nAbs conferring protection from infection because of its size and the length of the follow-up. Other limitations are the lack of assessment of the associations of the underlying disease and therapy with the vaccine-induced T-cell immunity as well as of individual therapeutic agents with the vaccine-induced antibody responses.

## Conclusions

The ImmunoVax comparative effectiveness study found substantial differences in waning of the nAb responses against 2019-nCoV vs the VOCs. The proportions of participants with positive nAbs and antibody titers were significantly lower in the responses against the Beta and Delta variants. Likewise, the durability of these responses against the Beta and Delta variants over 6 months was much shorter. Of note, the estimated durability in days of binding IgG anti-S antibodies surpassed by 4.0- to 9.0-fold that of nAbs against 2019-nCoV and the VOCs. Also of note, mRNA-1273 was associated with humoral responses of greater magnitude and longer duration than BNT162b2. Therefore, these findings may have important implications for personalizing the vaccination-boosting strategies to the underlying disease and informing the choice of the best vaccine for vulnerable populations.

## Notes

Supplement.
eMethods.
**eFigure 1.** Recruitment of Participants, Laboratory Testing, and Follow-up **eFigure 2.** Levels of Binding IgG Anti-S Antibodies at Month 1 After the Second Vaccine Dose **eFigure 3.** Proportion of Participants With Neutralizing Antibody Responses at Month 1 and 3 Post-Vaccination **eFigure 4.** Levels of Neutralizing Antibody Responses at Month 1 and 3 After the Second Vaccine Dose **eFigure 5.** Levels of Neutralizing Antibody Responses Following Vaccination With the mRNA-1273 or BNT162b2 **eFigure 6.** Proportion of Participants With Different Levels of Neutralizing Antibody Titers **eFigure 7.** Proportion of Participants With Neutralizing Antibodies Responses at Month 1, 3 and 6 After the Second Vaccination **eFigure 8.** Estimates of the Duration in Time of Binding Response at Month 6 After the Second Dose in the participants with solid cancers **eFigure 9.** Estimates of the Duration in Time of Neutralizing Response at Month 6 Since the Second Dose in the HC Participants **eFigure 10.** Estimates of the Duration in Time of Neutralizing Response at Month 6 Since the Second Dose in the Participants With Hematologic Cancer **eFigure 11.** Percentage of Participants Reporting Local and Systemic Reactions at V2 After administration of BNT162b2 or mRNA-1273 **eTable 1.** Baseline Characteristics and Type of Vaccine of the Study Groups **eTable 2.** Baseline Treatments of Immunocompromised Patients **eTable 3.** Neutralizing Antibody Responses at Month 1 and 3 Post-Vaccination **eTable 4.** Influence of Immunosuppressive Treatments on Binding and Neutralizing Antibodies at Month 1 Post Vaccination **eTable 5.** Univariable Linear Regression Models of Binding and Neutralizing Antibodies at Month 1, 3 and 6 After the Second Dose of Vaccine
eReferences.

## References

[1] Kuderer NM, Choueiri TK, Shah DP, ; COVID-19 and Cancer Consortium. Clinical impact of COVID-19 on patients with cancer (CCC19): a cohort study. Lancet. 2020;395(10241):1907-1918. doi:10.1016/S0140-6736(20)31187-9 32473681 PMC7255743

[2] Boyarsky BJ, Werbel WA, Avery RK, . Antibody response to 2-dose SARS-CoV-2 mRNA vaccine series in solid organ transplant recipients. JAMA. 2021;325(21):2204-2206. doi:10.1001/jama.2021.7489 33950155 PMC8100911

[3] Akiyama S, Hamdeh S, Micic D, Sakuraba A. Prevalence and clinical outcomes of COVID-19 in patients with autoimmune diseases: a systematic review and meta-analysis. Ann Rheum Dis. 2020;annrheumdis-2020-218946. 33051220 10.1136/annrheumdis-2020-218946

[4] Mato AR, Roeker LE, Lamanna N, . Outcomes of COVID-19 in patients with CLL: a multicenter international experience. Blood. 2020;136(10):1134-1143. doi:10.1182/blood.2020006965 32688395 PMC7472711

[5] Scarfò L, Chatzikonstantinou T, Rigolin GM, . COVID-19 severity and mortality in patients with chronic lymphocytic leukemia: a joint study by ERIC, the European Research Initiative on CLL, and CLL Campus. Leukemia. 2020;34(9):2354-2363. doi:10.1038/s41375-020-0959-x 32647324 PMC7347048

[6] Kates OS, Haydel BM, Florman SS, . COVID-19 in solid organ transplant: a multi-center cohort study. Clin Infect Dis. 2020. 32766815

[7] Polack FP, Thomas SJ, Kitchin N, ; C4591001 Clinical Trial Group. Safety and efficacy of the BNT162b2 mRNA Covid-19 vaccine. N Engl J Med. 2020;383(27):2603-2615. doi:10.1056/NEJMoa2034577 33301246 PMC7745181

[8] Baden LR, El Sahly HM, Essink B, ; COVE Study Group. Efficacy and safety of the mRNA-1273 SARS-CoV-2 vaccine. N Engl J Med. 2021;384(5):403-416. doi:10.1056/NEJMoa2035389 33378609 PMC7787219

[9] Mahil SK, Bechman K, Raharja A, . The effect of methotrexate and targeted immunosuppression on humoral and cellular immune responses to the COVID-19 vaccine BNT162b2: a cohort study. Lancet Rheumatol. 2021;3(9):e627-e637. doi:10.1016/S2665-9913(21)00212-5 34258590 PMC8266273

[10] Boyarsky BJ, Werbel WA, Avery RK, . Immunogenicity of a single dose of SARS-CoV-2 messenger RNA vaccine in solid organ transplant recipients. JAMA. 2021;325(17):1784-1786. doi:10.1001/jama.2021.4385 33720292 PMC7961463

[11] Herishanu Y, Avivi I, Aharon A, . Efficacy of the BNT162b2 mRNA COVID-19 vaccine in patients with chronic lymphocytic leukemia. Blood. 2021;137(23):3165-3173. doi:10.1182/blood.2021011568 33861303 PMC8061088

[12] Perry C, Luttwak E, Balaban R, . Efficacy of the BNT162b2 mRNA COVID-19 vaccine in patients with B-cell non-Hodgkin lymphoma. Blood Adv. 2021;5(16):3053-3061. doi:10.1182/bloodadvances.2021005094 34387648 PMC8362658

[13] Crombie JL, Sherman AC, Cheng CA, . Activity of mRNA COVID-19 vaccines in patients with lymphoid malignancies. Blood Adv. 2021;5(16):3062-3065. doi:10.1182/bloodadvances.2021005328 34387646 PMC8362656

[14] Moor MB, Suter-Riniker F, Horn MP, . Humoral and cellular responses to mRNA vaccines against SARS-CoV-2 in patients with a history of CD20 B-cell-depleting therapy (RituxiVac): an investigator-initiated, single-centre, open-label study. Lancet Rheumatol. 2021;3(11):e789-e797. doi:10.1016/S2665-9913(21)00251-4 34514436 PMC8423431

[15] Pegu A, O’Connell SE, Schmidt SD, ; mRNA-1273 Study Group. Durability of mRNA-1273 vaccine-induced antibodies against SARS-CoV-2 variants. Science. 2021;373(6561):1372-1377. doi:10.1126/science.abj4176 34385356 PMC8691522

[16] Davies NG, Abbott S, Barnard RC, ; CMMID COVID-19 Working Group; COVID-19 Genomics UK (COG-UK) Consortium. Estimated transmissibility and impact of SARS-CoV-2 lineage B.1.1.7 in England. Science. 2021;372(6538):eabg3055. doi:10.1126/science.abg3055 33658326 PMC8128288

[17] Sabino EC, Buss LF, Carvalho MPS, . Resurgence of COVID-19 in Manaus, Brazil, despite high seroprevalence. Lancet. 2021;397(10273):452-455. doi:10.1016/S0140-6736(21)00183-5 33515491 PMC7906746

[18] Cele S, Gazy I, Jackson L, ; Network for Genomic Surveillance in South Africa; COMMIT-KZN Team. Escape of SARS-CoV-2 501Y.V2 from neutralization by convalescent plasma. Nature. 2021;593(7857):142-146. doi:10.1038/s41586-021-03471-w 33780970 PMC9867906

[19] Garcia-Beltran WF, Lam EC, St Denis K, . Multiple SARS-CoV-2 variants escape neutralization by vaccine-induced humoral immunity. Cell. 2021;184(9):2372-2383.e9. doi:10.1016/j.cell.2021.03.013 33743213 PMC7953441

[20] Wang Z, Schmidt F, Weisblum Y, . mRNA vaccine-elicited antibodies to SARS-CoV-2 and circulating variants. Nature. 2021;592(7855):616-622. doi:10.1038/s41586-021-03324-6 33567448 PMC8503938

[21] Chen RE, Zhang X, Case JB, . Resistance of SARS-CoV-2 variants to neutralization by monoclonal and serum-derived polyclonal antibodies. Nat Med. 2021;27(4):717-726. doi:10.1038/s41591-021-01294-w 33664494 PMC8058618

[22] Wang P, Nair MS, Liu L, . Antibody resistance of SARS-CoV-2 variants B.1.351 and B.1.1.7. Nature. 2021;593(7857):130-135. doi:10.1038/s41586-021-03398-2 33684923

[23] Stamatatos L, Czartoski J, Wan YH, . mRNA vaccination boosts cross-variant neutralizing antibodies elicited by SARS-CoV-2 infection. Science. 2021;25:eabg9175. doi:10.1126/science.abg9175 33766944 PMC8139425

[24] Lucas C, Vogels CBF, Yildirim I, ; Yale SARS-CoV-2 Genomic Surveillance Initiative. Impact of circulating SARS-CoV-2 variants on mRNA vaccine-induced immunity. Nature. 2021;600(7889):523-529. doi:10.1038/s41586-021-04085-y 34634791 PMC9348899

[25] Bergwerk M, Gonen T, Lustig Y, . Covid-19 breakthrough infections in vaccinated health care workers. N Engl J Med. 2021;385(16):1474-1484. doi:10.1056/NEJMoa2109072 34320281 PMC8362591

[26] Abu-Raddad LJ, Chemaitelly H, Ayoub HH, . Association of prior SARS-CoV-2 infection with risk of breakthrough infection following mRNA vaccination in Qatar. JAMA. 2021;326(19):1930-1939. doi:10.1001/jama.2021.19623 34724027 PMC8561432

[27] McDade TW, Demonbreun AR, Sancilio A, Mustanski B, D’Aquila RT, McNally EM. Durability of antibody response to vaccination and surrogate neutralization of emerging variants based on SARS-CoV-2 exposure history. Sci Rep. 2021;11(1):17325. doi:10.1038/s41598-021-96879-3 34462501 PMC8405730

[28] Levin EG, Lustig Y, Cohen C, . Waning immune humoral response to BNT162b2 Covid-19 vaccine over 6 months. N Engl J Med. 2021;385(24):e84. doi:10.1056/NEJMoa2114583 34614326 PMC8522797

[29] Chemaitelly H, Tang P, Hasan MR, . Waning of BNT162b2 vaccine protection against SARS-CoV-2 infection in Qatar. N Engl J Med. 2021;385(24):e83. doi:10.1056/NEJMoa2114114 34614327 PMC8522799

[30] Zhong D, Xiao S, Debes AK, . Durability of antibody levels after vaccination with mRNA SARS-CoV-2 vaccine in individuals with or without prior infection. JAMA. 2021;326(24):2524-2526. doi:10.1001/jama.2021.19996 34724529 PMC8561429

[31] Eliakim-Raz N, Massarweh A, Stemmer A, Stemmer SM. Durability of response to SARS-CoV-2 BNT162b2 vaccination in patients on active anticancer treatment. JAMA Oncol. 2021;7(11):1716-1718. doi:10.1001/jamaoncol.2021.4390 34379092 PMC8358809

[32] Hoffmann M, Arora P, Groß R, . SARS-CoV-2 variants B.1.351 and P.1 escape from neutralizing antibodies. Cell. 2021;184(9):2384-2393.e12. doi:10.1016/j.cell.2021.03.036 33794143 PMC7980144

[33] Shen X, Tang H, Pajon R, . Neutralization of SARS-CoV-2 variants B.1.429 and B.1.351. N Engl J Med. 2021;384(24):2352-2354. doi:10.1056/NEJMc2103740 33826819 PMC8063884

[34] Berger ML, Mamdani M, Atkins D, Johnson ML. Good research practices for comparative effectiveness research: defining, reporting and interpreting nonrandomized studies of treatment effects using secondary data sources: the ISPOR Good Research Practices for Retrospective Database Analysis Task Force Report–Part I. Value Health. 2009;12(8):1044-1052. doi:10.1111/j.1524-4733.2009.00600.x 19793072

[35] Fenwick C, Turelli P, Pellaton C, . A high-throughput cell- and virus-free assay shows reduced neutralization of SARS-CoV-2 variants by COVID-19 convalescent plasma. Sci Transl Med. 2021;13(605):eabi8452. doi:10.1126/scitranslmed.abi8452 34257144 PMC9835890

[36] Fenwick C, Croxatto A, Coste AT, . Changes in SARS-CoV-2 spike versus nucleoprotein antibody responses impact the estimates of infections in population-based seroprevalence studies. J Virol. 2021;95(3):e01828-20. doi:10.1128/JVI.01828-20 33144321 PMC7925109

[37] Collier AY, Yu J, McMahan K, . Differential kinetics of immune responses elicited by Covid-19 vaccines. N Engl J Med. 2021;385(21):2010-2012. doi:10.1056/NEJMc2115596 34648703 PMC8531985

[38] Liu Y, Liu J, Xia H, . Neutralizing activity of BNT162b2-elicited serum. N Engl J Med. 2021;384(15):1466-1468. doi:10.1056/NEJMc2102017 33684280 PMC7944950

[39] Addeo A, Shah PK, Bordry N, . Immunogenicity of SARS-CoV-2 messenger RNA vaccines in patients with cancer. Cancer Cell. 2021;39(8):1091-1098.e2. doi:10.1016/j.ccell.2021.06.009 34214473 PMC8218532

