# Supplementary material for: Humoral Responses Against Variants of Concern by COVID-19 mRNA Vaccines in Immunocompromised Patients
Source: JAMA Oncol. 2022 Mar 10;8(5):e220446. doi: 10.1001/jamaoncol.2022.0446 (PMC8914885; doi:10.1001/jamaoncol.2022.0446)
Supplement: Supplement. — eMethods. eFigure 1. Recruitment of Participants, Laboratory Testing, and Follow-up eFigure 2. Levels of Binding IgG Anti-S Antibodies at Month 1 After the Second Vaccine Dose eFigure 3. Proportion of Participants With Neutralizing Antibody Responses at Month 1 and 3 Post-Vaccination eFigure 4. Levels of Neutralizing Antibody Responses at Month 1 and 3 After the Second Vaccine Dose eFigure 5. Levels of Neutralizing Antibody Responses Following Vaccination With the mRNA-1273 or BNT162b2 eFigure 6. Proportion of Participants With Different Levels of Neutralizing Antibody Titers eFigure 7. Proportion of Participants With Neutralizing Antibodies Responses at Month 1, 3 and 6 After the Second Vaccination eFigure 8. Estimates of the Duration in Time of Binding Response at Month 6 After the Second Dose in the participants with solid cancers eFigure 9. Estimates of the Duration in Time of Neutralizing Response at Month 6 Since the Second Dose in the HC Participants eFigure 10. Estimates of the Duration in Time of Neutralizing Response at Month 6 Since the Second Dose in the Participants With Hematologic Cancer eFigure 11. Percentage of Participants Reporting Local and Systemic Reactions at V2 After administration of BNT162b2 or mRNA-1273 eTable 1. Baseline Characteristics and Type of Vaccine of the Study Groups eTable 2. Baseline Treatments of Immunocompromised Patients eTable 3. Neutralizing Antibody Responses at Month 1 and 3 Post-Vaccination eTable 4. Influence of Immunosuppressive Treatments on Binding and Neutralizing Antibodies at Month 1 Post Vaccination eTable 5. Univariable Linear Regression Models of Binding and Neutralizing Antibodies at Month 1, 3 and 6 After the Second Dose of Vaccine eReferences. [file jamaoncol-e220446-s001.pdf]

Supplemental Online Content

Obeid M, Suffiotti M, Pellaton C, et al. Humoral responses against variants of concern by COVID-19 mRNA vaccines in immunocompromised patients. *JAMA Oncol*. Published online March 10, 2022. doi:10.1001/jamaoncol.2022.0446

eMethods.

eFigure 1. Recruitment of Participants, Laboratory Testing, and Follow-up

eFigure 2. Levels of Binding IgG Anti-S Antibodies at Month 1 After the Second Vaccine Dose

eFigure 3. Proportion of Participants With Neutralizing Antibody Responses at Month 1 and 3 Post-Vaccination

eFigure 4. Levels of Neutralizing Antibody Responses at Month 1 and 3 After the Second Vaccine Dose

eFigure 5. Levels of Neutralizing Antibody Responses Following Vaccination With the mRNA-1273 or BNT162b2

eFigure 6. Proportion of Participants With Different Levels of Neutralizing Antibody Titers

eFigure 7. Proportion of Participants With Neutralizing Antibodies Responses at Month 1, 3 and 6 After the Second Vaccination

eFigure 8. Estimates of the Duration in Time of Binding Response at Month 6 After the Second Dose in the participants with solid cancers

eFigure 9. Estimates of the Duration in Time of Neutralizing Response at Month 6 Since the Second Dose in the Hematologic Cancer Participants

eFigure 10. Estimates of the Duration in Time of Neutralizing Response at Month 6 Since the Second Dose in the Participants With Hematologic Cancer

eFigure 11. Percentage of Participants Reporting Local and Systemic Reactions at V2 After administration of BNT162b2 or mRNA-1273

eTable 1. Baseline Characteristics and Type of Vaccine of the Study Groups

eTable 2. Baseline Treatments of Immunocompromised Patients

eTable 3. Neutralizing Antibody Responses at Month 1 and 3 Post-Vaccination

eTable 4. Influence of Immunosuppressive Treatments on Binding and Neutralizing Antibodies at Month 1 Post Vaccination

eTable 5. Univariable Linear Regression Models of Binding and Neutralizing Antibodies at Month 1, 3 and 6 After the Second Dose of Vaccine

eReferences.

© 2022 Obeid M et al. *JAMA Oncology.*

This supplemental material has been provided by the authors to give readers additional information about their work.

© 2022 Obeid M et al. *JAMA Oncology.*

eMethods

1.1 Serologic assays and procedures

The levels of binding IgG anti-Spike (S) and anti-Nucleocapdide (N) antibodies were determined using a Luminex-based assay recently developed in our laboratory and a ratio >6.0 corresponds to the cutoff for diagnostic positivity[1]. Neutralizing antibody responses were assessed using a cell- and virus-free surrogate neutralization assay recently developed in our laboratory[2]. In this assay, neutralizing antibodies block the ability of fluorescent angiotensin converting enzyme 2 (ACE2) molecules from binding to recombinant SARS-CoV-2 Spike protein trimers. The assay achieved 96.7% sensitivity and 100% specificity in cross-validation studies with a gold standard, live virus cell-based assay and could be multiplexed to quantify responses against SARS-CoV-2 VOCs in one test. Only sera with positive binding IgG anti-S antibodies were evaluated for neutralizing antibodies. The neutralizing activity was measured as IC50 dilutions of the serum corresponding to the serum dilution inhibiting 50% of the Spike/ACE2 binding. On the basis of the cross-validation with the live virus cell-based assay, an IC50 >50 corresponds to the cutoff for a positive diagnostic test[2] and thus IC50 titers <50 were considered as a negative response. Therefore, the threshold for neutralizing activity was set at 50: <50: negative neutralizing activity, ≥ 50 to <100: weak neutralizing activity, ≥ 100 to <150: moderate neutralizing activity, ≥ 150: good neutralizing activity.

1.2 IgG ratio transformation in unit/ml (WHO units)

In order to transform IgG ratio values into the WHO unit/ml, we used a robust linear regression model (rlm function from MASS package in R) on 298 samples with paired measurements using ($\log_{10}$) unit/ml measurement as response and ($\log_{10}$) ratios as covariate. Then, we applied the resulting model on all ($\log_{10}$) IgG ratio values to transform them into ($\log_{10}$) unit/ml.

© 2022 Obeid M et al. *JAMA Oncology.*

Estimated model's parameters

Parameter	Regression coefficient
Intercept	-0.6108069
Slope	2.0072882

1.3 ISPOR guideline

This study follows the ISPOR reporting guideline[34] for comparative effectiveness research to improve effectiveness assessment in the form of nonrandomized studies using secondary databases. The rationale for the observational study were explicitly stated. There are no direct comparative data on the effectiveness of Covid-19 mRNA vaccines in immunocompromised patients and healthy participants. The research questions and hypotheses addressed are relevant and the add value of this study to the pandemic in immunocompromised individuals is important. The research methodology and serological assays have been standardized and automated to guarantee reliable, reproducible and homogeneous results with the minimum of technical error. A narrative description is included in the methods section. The study design and data-analysis were appropriate with adequate numbers of patients to yield sufficient statistical power for the primary analyses. The study design is also appropriate to address the study hypotheses/questions and included two groups of participants vaccinated with BNT162b2 or mRNA-1273. Standardized reporting data system and careful interpretation of results were implemented. The interpretation was conducted with sophisticated statistical methods to improve causal inference of age, gender and treatment effects.

© 2022 Obeid M et al. *JAMA Oncology.*

eFigure 1. Recruitment of participants, Laboratory testing, and Follow-up.
This is a prospective longitudinal study of immunocompromised patients and of health care workers as group of control. Participants received two doses of BNT162b2 or mRNA-1273 vaccines. Between January 14 and December 18, 2021, the participants were monitored for 6 months after the 2nd dose of vaccine. Seroconversion to SARS-CoV-2 Spike (S) protein and neutralizing antibodies were tested before vaccination and longitudinally at Week 1, Month 1, 3, and 6 following the 2nd vaccine dose. All participants underwent to 3-4 serologic assays.

1

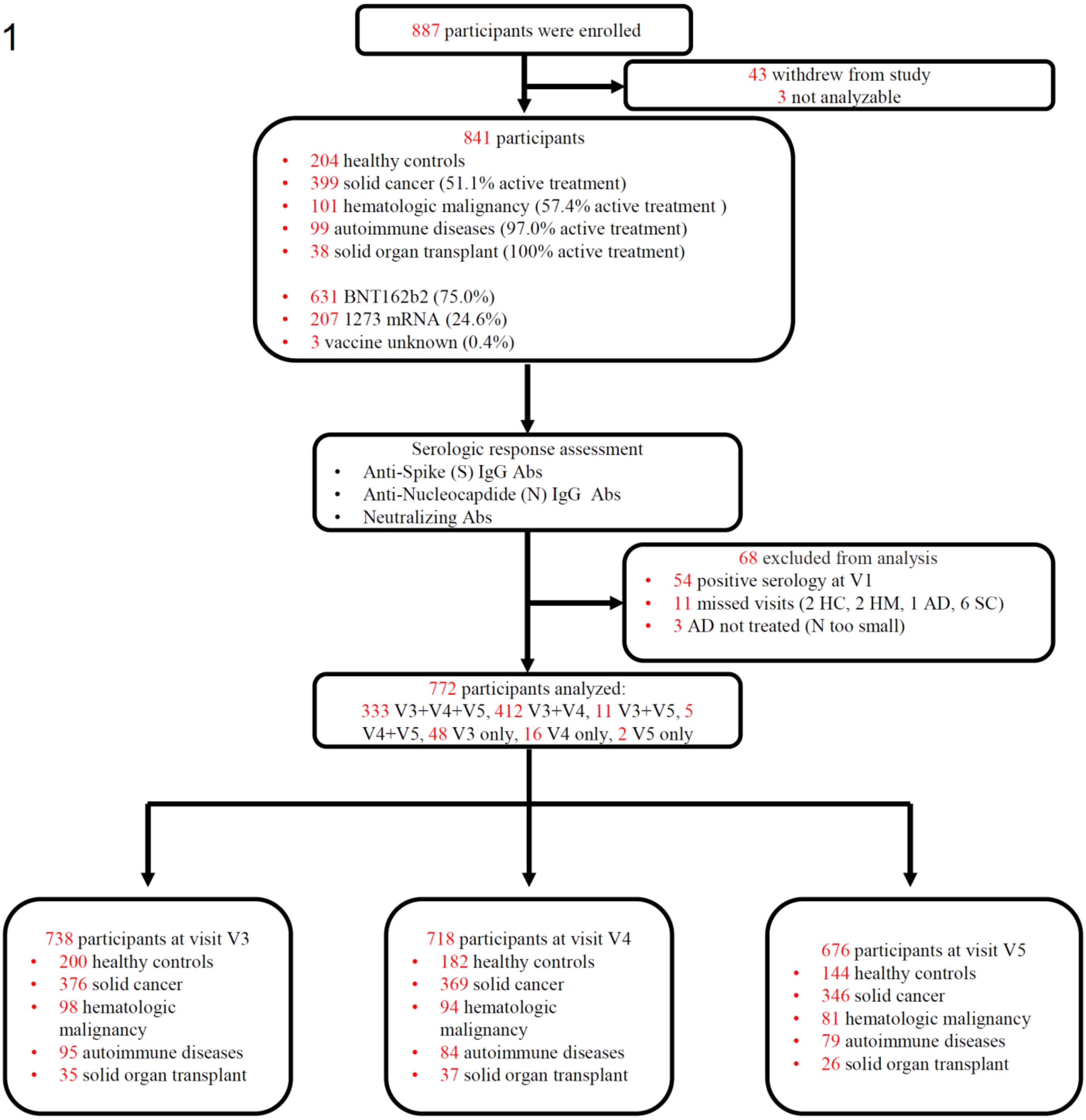

© 2022 Obeid M et al. *JAMA Oncology.*

eTable 1. Baseline characteristics and type of vaccine of the study groups

Characteristics	N	%
Study groups<br>age 58.0±14.9 (mean±SD)<br>311 Male, 530 Female	841	100%
Healthy control participants<br>age 45.9±12.0<br>60 Male, 144 Female	204	24.3%
Solid cancers<br>age 63.8±12.3<br>139 Male, 260 Female	399	47.4%
Hemato. cancers<br>age 63.2±13.8<br>55 Male, 46 Female	101	12.0%
AD diseases<br>age 52.6±15.8<br>31 Male, 68 Female	99	11.8%
SOT patients<br>age 60.9±12.4<br>26 Male, 12 Female	38	4.5%
Solid cancers		
Breast	173	43.4%
Thoracic	64	16.0%
Genitourinary	48	12.0%
Gastrointestinal	45	11.3%
Skin	24	6.0%
Sarcoma	12	3.0%
Brain	9	2.3%
Hepatic	6	1.5%
Lung	6	1.5%
Neuroendocrine	5	1.3%
Renal	4	1.0%
Head and neck	3	0.8%
Haematological malignancies		
Multiple myeloma	23	22.8%
Other lymphoma	12	11.9%
Diffuse large B cell lymphoma	11	10.9%
AML	9	8.9%
CML	8	7.9%
Hodgkin lymphoma	8	7.9%
Other leukemia	7	6.9%
Follicular lymphoma	7	6.9%
CLL	6	5.9%
Waldenstrom macroglobulinemia	4	4.0%
MDS or aplastic anemia	4	4.0%
MGRS	1	1.0%

© 2022 Obeid M et al. *JAMA Oncology.*

Other disorders	1	1.0%
Autoimmune diseases		
Primary immunodeficiency	15	15.2%
Vasculitis	14	14.1%
Sjoegren's syndrome	14	14.1%
SLE	12	12.1%
Sarcoidosis	9	9.1%
Other	8	8.1%
Autoinflammatory diseases	6	6.1%
Inflammatory cardiomyopathy	6	6.1%
Uveitis	5	5.1%
Behcet's	4	4.0%
Myasthenia gravis	2	2.0%
Undifferentiated or mixed connective tissue disease	2	2.0%
Systemic sclerosis	2	2.0%
Type of organ transplant		
Kidney	27	71.1%
Liver	7	18.4%
Multiorgan	3	7.9%
Lung	1	2.6%
Type of vaccine (3 unknown, N=838)		
BNT162b2	631	75.3%
mRNA-1273	207	24.7%

HC: healthy controls; SC: solid cancers; HM: haematological malignances; AD: autoimmune diseases; SOT: solid organ transplants

© 2022 Obeid M et al. *JAMA Oncology.*

eTable 2. Baseline treatments of immunocompromised patients

	N	%
**Type of cancer treatment**		
• Hormonal therapy	112	17.6%
• Chemotherapy	36	5.7%
• Immune checkpoint inhibitor ICI	23	3.6%
• Tyrosine kinase inhibitor TKI	21	3.3%
• Immunomodulator drugs IMIDs	11	1.7%
• anti-CD20 antibody therapy (<365 days) (375 mg/m2)	11	1.7%
• Chemotherapy + Immune checkpoint inhibitor	7	1.1%
• Anti-HER2 antibody therapy	6	0.9%
• Anti-CD38 antibody therapy	4	0.6%
• BCL-2 inhibitor	3	0.5%
• PIs + IMIDs	3	0.5%
• VEGF inhibitor	3	0.5%
• BTK inhibitor	3	0.5%
• PARP inhibitor	2	0.3%
• Antibody-drug conjugate	2	0.3%
• AR-targeted therapy	2	0.3%
• RANKL inhibitors	1	0.2%
• Protease inhibitor PIs	1	0.2%
• anti-CD20 therapy (> 1 year) (375 mg/m2)	1*	0.2%*
**Type of immunosuppressant drugs**		
• CNI+ IMDHIs	34	5.3%
• anti-CD20 antibody therapy (<365 days) (1g, 0.5g, 025g)	16	2.5%
• bDMARD + cs DMARD	15	2.4%
• bDMARD	15	2.4%
• csDMARD	12	1.9%
• IMDHI + cs or b DMARD	10	1.6%
• Janus kinase inhibitors	5	0.8%
• IMDHI	5	0.8%
• CS	4	0.6%
• mTOR inhibitor	3	0.5%
• CNI	2	0.3%
• anti-CD20 therapy (> 1 year) (1g, 0.5g, 025g)	2*	0.3%*
• anti-complement therapy	1	0.2%
• CNI + cs or bDMARD + anti-CD20 therapy (> 1 year) (1g,0.5g, 025g)	1	0.2%
**Other treatments**		
• IVIG	17 (+11* in combination)	2.7 % (1.7%*)
**No treatment**		
• SC (N=399, 49.9%)	199	31.2%
• HM (N=101, 43.6%)	44	6.9%
• AD (N=99, 3.0%)	3	0.5%

*not included in the total

AR, androgen receptor; BCL-2, B cell lymphoma 2; BTK, Bruton's tyrosine kinase; bDMARD biological disease-modifying antirheumatic drugs, csDMARD conventional synthetic disease-modifying antirheumatic drugs ,

© 2022 Obeid M et al. *JAMA Oncology.*

RANKL RANK ligand, mTOR, mammalian target of rapamycin; VEGF, vascular endothelial growth factor, HCQ hydroxychloroquine, MTX methotrexate, IVIG intravenous immunoglobulin, IMDHIs Inosine monophosphate dehydrogenase inhibitors, CNI Calcineurin inhibitor drugs, a bDMARD: Infliximab, Adalimumab, Abatacept, tocilizumab, Mepolizumab, Anakinra and b csDMARD: Methotrexate, Hydroxychloroquine, Colchicine.

© 2022 Obeid M et al. *JAMA Oncology.*

eFigure 2. Levels of binding IgG anti-S antibodies at month 1 after the 2nd vaccine dose. **A.** Percentage of participants with positive diagnostic for binding IgG anti-S antibodies. **B.** Titers, i.e. units/ml, of binding IgG anti-S antibodies. **C.** Titers of binding IgG anti-S antibodies in participants vaccinated with the mRNA-1273 or the BNT162b2 vaccines. All the study populations are shown. Resulting p-values were adjusted for multiple testing using the False Discovery Rate (FDR) approach of Benjamini-Hochberg. The titers in SOT, AD and treated HM were significantly lower (*P*<0.001) compared to HC

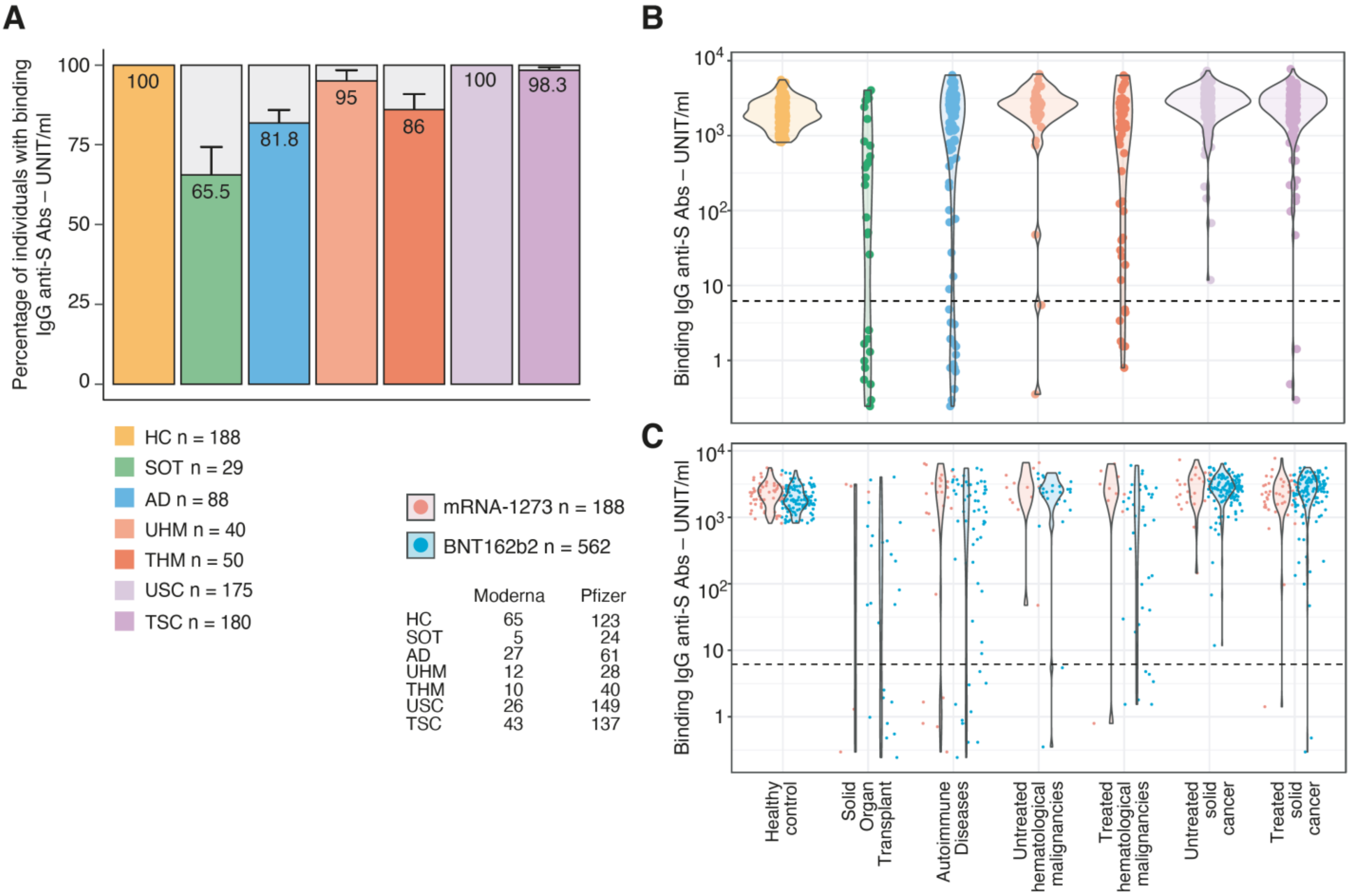

© 2022 Obeid M et al. *JAMA Oncology.*

eFigure 3. Proportion of participants with neutralizing antibodies responses at month 1 and 3 post-vaccination.
Proportion of participants positive for neutralizing antibody at Month 1 and 3 in healthy control (**A**), solid organ transplant (**B**) and autoimmune diseases (**C**). Neutralizing antibody responses were measured against the original 2019nCoV and the different VOCs. Data are expressed as IC50 dilutions.

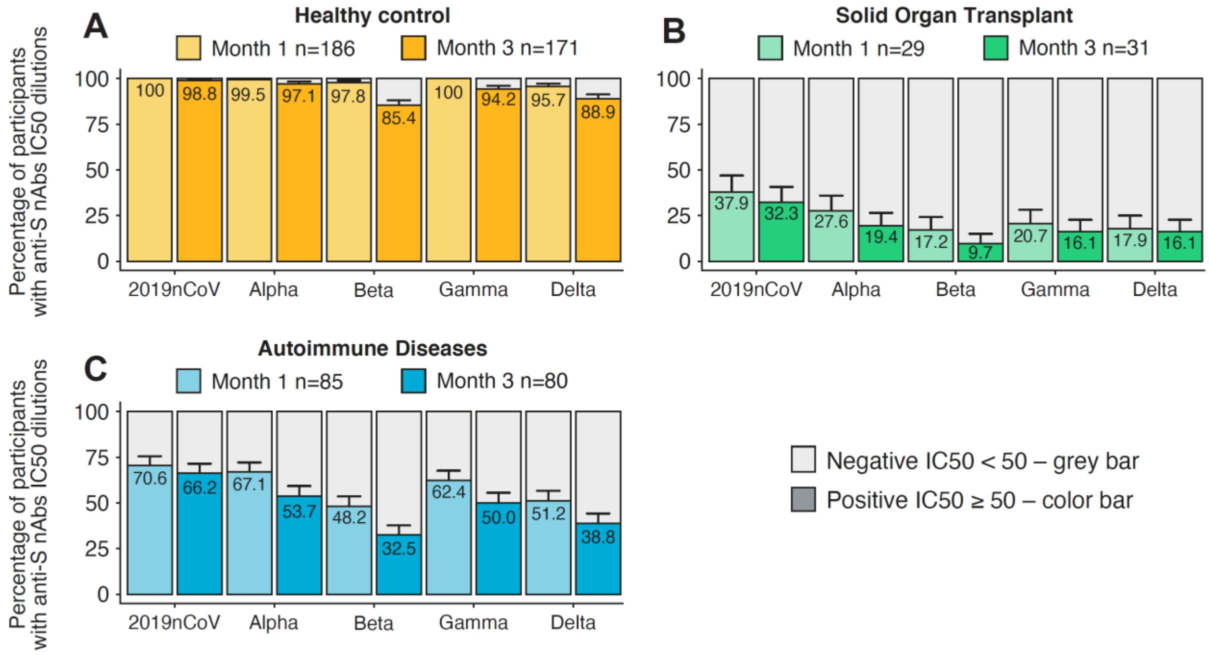

© 2022 Obeid M et al. *JAMA Oncology.*

eFigure 4. Levels of neutralizing antibody responses at month 1 and 3 after the 2nd vaccine dose.

IC50 titers of neutralizing antibodies at Month 1 and 3 in healthy control (**A**), solid organ transplant (**B**) and autoimmune diseases. The dotted line indicates the threshold positivity of the assay, i.e. IC50 >50 dilutions. IC50 dilutions were log10 transformed for analysis. Resulting p-values were adjusted for multiple testing using the False Discovery Rate (FDR) approach of Benjamini-Hochberg.
P*<0.05; ***P*<0.01; * *P*<0.001

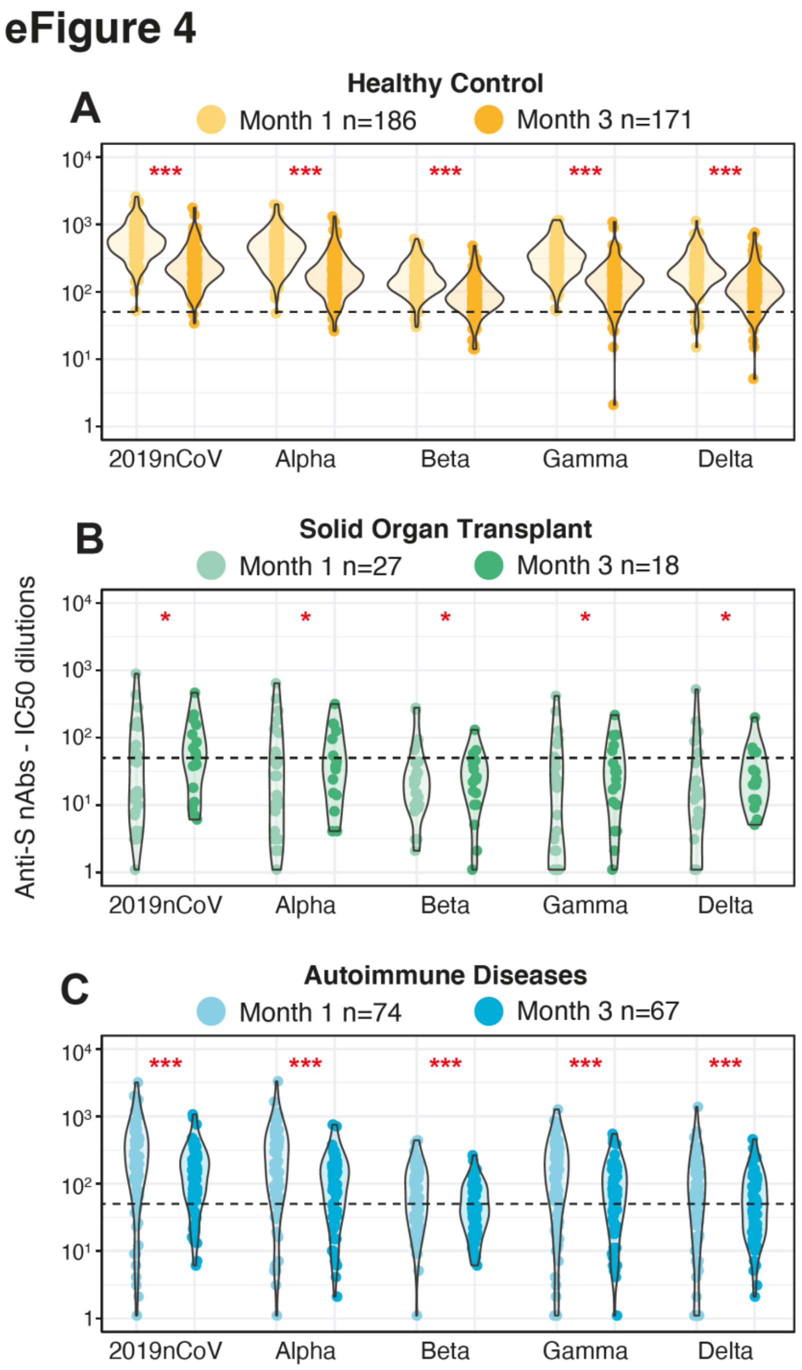

© 2022 Obeid M et al. *JAMA Oncology.*

eTable 3. Neutralizing antibody responses at Month 1 and 3 post-vaccination

	Month 1			Month 3		
**Healthy Controls**	**Median**	**95% CI low**	**95% CI high**	**Median**	**95% CI low**	**95% CI high**
**2019nCoV**	531.9	483.1	584.4	219.4	199.5	237.4
**Alpha**	418.7	384.8	459.3	167.8	143.7	179.5
**Beta**	142.3	130.2	153.4	83.5	75.2	87.4
**Gamma**	312.8	280.8	337.9	135.8	122.7	150.5
**Delta**	197.1	183.2	216.4	102.4	95.3	115.1

**Solid Organ Transplant**	Month 1			Month 3		
	**Median**	**95% CI low**	**95% CI high**	**Median**	**95% CI low**	**95% CI high**
**2019nCoV**	16.5	8.5	68.1	54.4	18.4	86.5
**Alpha**	13.6	2.5	43.5	37.6	13.6	53.6
**Beta**	22.4	10.4	30.4	24.9	10.5	39.0
**Gamma**	6.6	0.7	27.8	27.6	9.5	41.9
**Delta**	10.2	3.5	16.5	22.3	11.1	33.4

**Untreated Hematogical Cancers**	Month 1			Month 3		
	**Median**	**95% CI low**	**95% CI high**	**Median**	**95% CI low**	**95% CI high**
**2019nCoV**	490.4	290.5	707.3	341.7	163.4	456.4
**Alpha**	360.0	221.7	551.9	216.8	132.7	353.8
**Beta**	121.2	98.0	153.3	97.3	71.4	121.0
**Gamma**	230.5	148.6	387.9	156.6	112.0	258.9
**Delta**	178.5	129.2	253.1	111.0	79.1	200.4

**Treated Hematogical Cancers**	Month 1			Month 3		
	**Median**	**95% CI low**	**95% CI high**	**Median**	**95% CI low**	**95% CI high**
**2019nCoV**	255.4	136.2	431.3	134.3	88.2	176.1
**Alpha**	217.5	105.8	318.7	90.8	59.7	122.8
**Beta**	73.3	42.5	112.2	50.3	34.5	60.4
**Gamma**	134.8	86.9	189.0	83.6	40.9	108.6
**Delta**	77.1	36.1	143.3	60.6	29.4	94.5

**Autoimmune Diseases**	Month 1			Month 3		
	**Median**	**95% CI low**	**95% CI high**	**Median**	**95% CI low**	**95% CI high**
**2019nCoV**	208.3	164.4	373.5	144.3	82.1	176.2
**Alpha**	174.0	94.6	271.9	84.0	49.5	145.9
**Beta**	61.3	44.3	85.4	43.2	30.4	55.3
**Gamma**	123.7	77.9	165.7	66.5	41.8	101.8
**Delta**	64.4	36.4	80.5	48.3	26.3	60.1

**Untreated Solid Cancers**	Month 1			Month 3		
	**Median**	**95% CI low**	**95% CI high**	**Median**	**95% CI low**	**95% CI high**
**2019nCoV**	465.1	406.4	529.3	172.2	154.5	194.2
**Alpha**	322.0	270.5	349.7	120.7	112.5	142.0
**Beta**	128.4	104.5	133.5	69.5	57.1	76.2
**Gamma**	259.9	218.8	297.0	104.7	93.8	116.9
**Delta**	163.5	142.4	185.1	78.0	62.4	90.4

**Treated Solid Cancers**	Month 1			Month 3		
	**Median**	**95% CI low**	**95% CI high**	**Median**	**95% CI low**	**95% CI high**
**2019nCoV**	474.9	401.2	551.5	194.7	152.6	226.6
**Alpha**	330.8	262.5	401.9	138.6	109.0	168.3
**Beta**	118.0	104.2	137.5	73.9	63.5	85.6
**Gamma**	251.7	207.6	311.7	109.8	94.5	129.9
**Delta**	172.3	134.3	188.5	91.2	75.0	106.5

© 2022 Obeid M et al. *JAMA Oncology.*

eTable 4. Influence of immunosuppressive treatments on binding and neutralizing antibodies at Month 1 post vaccination

			Seroconversion										Spike-ACE2 IC50 dilution						
			IgG [U/ml]			n2019CoV			Alpha			Beta			Gamma			Delta	
		N	Median	2.50%	97.50%	Median	2.50%	97.50%	Median	2.50%	97.50%	Median	2.50%	97.50%	Median	2.50%	97.50%	Median	2.50%	97.50%
Age	< 65	453	2227.296	2109.68	2387.442	484	447	512	378	340	405	131	124	140	273	248	296	177	164	189
	≥ 65	261	2604.489	2432.09	2798.834	363	281	413	227	189	263	90	79	100	189	161	208	115.5	92	136
Sex	Female	449	2402.277	2280.06	2563.391	480	447	531	369	330	405	130	120	138	274	246	299	177	162	189
	Male	265	2311.532	2026.98	2502.359	378	314	425	260	222	319	102	91	113	200	181	230	132	105	153
Type of cancer treatment (HM=38, SC=174)																				
	Hormonal therapy	101	2804.2	2457.1	3191.4	464	381	619	356	239	439	122	97	146	246	206	331	169	133	217
	Chemotherapy	28	2248.9	1298.4	2548.1	474.5	178	634	325	105	481	113.5	46	204	194	97	322	169	72	223
	Immune checkpoint inhibitor ICI	20	2454.8	1981.8	2852.6	550	374	1517	425.5	291	898	162.5	118	308	322	214	684	208.5	174	492
	Tyrosine kinase inhibitor TKI	17	2719.2	2151.7	3266.1	489	306	1544	319	213	954	106	85	300	220	156	704	155	80	422
	Immunomodulator drugs IMIDs	8	2680.6	131.6	5020.3	323.5	14	551	246.5	8	436	101.5	11	195	160.5	7	311	129	16	162
	anti-CD20 antibody therapy (<365 days) (375 mg/m2)	6	352.3	-0.5	1717.0	46	2	149	26	0	121	14.5	10	43	8.5	1	109	13	2	60
	Chemotherapy + Immune checkpoint inhibitor	6	3293.1	253.1	3863.3	394	42	1565	206	20	1054	70	10	317	192	16	594	58.5	4	499
	AntiHER2 antibody therapy	5	2232.1			492			326			125			288			181		
	AntiCD38 antibody therapy	4	178.8			23			16.5			17.5			15.5			6.5		
	PIs + IMIDs	3	759.4			107			61			39			22			9		
	BCL2 inhibitor	2	1129.7			230			192			90.5			127.5			109.5		
	BTK inhibitor	2	1163.4			171.5			109.5			41			83			43.5		
	Antibodydrug conjugate	2	1481.7			1984.5			1323			302.5			544			584		
	ARtargeted therapy	2	2612.0			679.5			237.5			85			351.5			43		
	PARP inhibitor	2	1309.9			2658			2188.5			701			1289			1214.5		
	VEGF inhibitor	2	2100.7			1057.5			366			92			547			88.5		
	Protease inhibitor PIs	1	3112.0			542			425			207			400			197		
	RANKL inhibitors	1	2814.9			640			260			48			391			31		
Immunosuppressive therapy (SOT=27, HM=2, AD=62)																				
	CNI+ IMDHIs	25	80.1	2.2	441.0	15	8	58	12	4	43	20	11	28	4	1	23	8	3	16
	bDMARD	10	2709.3	842.1	3447.7	192.5	116	487	152	70	418	59	27	183	93	65	315	62.5	4	174
	antiCD20 antibody therapy (<365 days) (1g, 0.5g, 025g)	10	155.4	-0.3	2151.7	30	3	255	18	1	196	18	9	47	13	1	160	8	1	24
	bDMARD + cs DMARD	9	1990.8	790.2	3483.4	196	132	413	151	90	338	62	42	171	125	46	296	72	30	204
	IMDHI + cs or b DMARD	9	2432.1	210.2	3248.8	113	41	460	94	34	386	34	15	128	85	26	241	28	13	140
	csDMARD	6	1594.0	877.6	3394.5	469.5	225	1026	313	140	629	115.5	80	197	235	123	372	165.5	90	350
	bDMARD + cs DMARD and IVIG	4	3237.0			386			429.5			98.5			173.5			56		
	IMDHI	4	1231.1			95.5			59.5			27.5			45.5			37.5		
	CS	3	871.7			180			152			59			116			70		
	mTOR inhibitor	3	2939.6			435			381			260			249			175		
	Janus kinase inhibitors and IVIG	2	688.4			64			56.5			29			43.5			21.5		
	Janus kinase inhibitors	2	270.2			102			58.5			25.5			34			42		
	CNI + cs or bDMARD + anti-CD20 therapy (> 1 year) (1g, 0.5g, 025g)	1	5230.4			835			597			126			419			279		
	CS + anti-CD20 therapy (> 1 year) (1g, 0.5g, 025g)	1	1486.7			2012			1653			304			867			490		
	csDMARD and IVIG	1	5020.3			451			453			148			296			132		
	Tyrosine kinase inhibitor TKI	1	3237.3			1149			844			194			688			355		
Other Treatments (HM=2, AD=12)	IVIG	13	2950.6	26.5	4079.9	445	26	775	378	16	666	120	20	207	247	12	443	139	16	231
	anti-CD20 therapy (> 1 year) (375 mg/m2) and IVIG	1	11.7			132			43			42			31			3		

© 2022 Obeid M et al. *JAMA Oncology.*

eLegend Figure 3. For example. at 1 month after vaccination, the IC50 titers against 2019nCoV were significantly lower in participants with solid organ transplants (median 16.5, 95% CI 8.5-68.1; P<0.001), autoimmune diseases (median 208.3, 95% CI 164.4-373.5; P<0.05), treated hematologic cancers (median 255.4, 95% CI 136.2-431.3; P<0.05) and untreated solid cancers (median 465.1, 95% CI 406.4-529.3; P<0.05) as compared with healthy controls (median 531.9, 95% CI 483.1-584.4, untreated hematological cancers (median 490.4, 95% CI 290.5-707.3 and treated solid cancers (median 475.9, 95% CI 401.2-551.2.
Similarly, the IC50 titers against the Delta variant were significantly lower in participants with solid organ transplants (median 10.2, 95% CI (3.5-16.5); P<0.001), autoimmune diseases (median 64.4, 95% CI (36.4-80.5); P<0.001), treated hematologic cancers (median 77.1, 95% CI (36.1-143.3); P<0.001) as compared with healthy controls (median 197.1, 95% CI (183.2-216.4), untreated solid cancers (median 163.5, 95% CI (142.4-185.1), treated solid cancers (median 172.3, 95 CI (134.3-188.5) and untreated hematological cancers (median 178.5, 95% CI (129.2-253.1) (eTable 3 in the Supplement.
At 3 months, in the untreated hematological cancers, the IC50 titers between the two vaccines were significantly different for the 2019-nCoV (*P* = 0.029) and all the other VOCs (Alpha *P* = 0.040, Beta *P* = 0.042, Gamma *P* = 0.045, and Delta *P* = 0.028). In the treated hematological cancers significant differences in IC50 titers were only observed for the 2019-nCoV (*P* = 0.045). In the untreated solid cancers, the IC50 titers between the two vaccines were significantly different for the 2019-nCoV (*P* = 0.004) and all the other VOCs (Alpha *P* = 0.013, Beta *P* = 0.029, Gamma *P* = 0.040, and Delta *P* = 0.004). Similarly, in the treated solid cancers, the IC50 titers between the two vaccines were significantly different for the 2019-nCoV (*P* = 0.001) and all the other VOCs (Alpha *P* = 0.001, Beta *P* = 0.002, Gamma *P* = 0.013, and Delta *P* < 001).

eFigure 5. Levels of neutralizing antibody responses following vaccination with the mRNA-1273 or BNT162b2.

IC50 titers of neutralizing antibodies in healthy control (**A**), solid organ transplant (**B**) and autoimmune diseases (**C)** study populations vaccinated with either the mRNA-1273 or the BNT162b2 vaccines. IC50 dilutions were log10 transformed for analysis. At 1 month, in the healthy controls the IC50 titers between the two vaccines were significantly different for the 2019-nCoV (*P* = 0.005), the Alpha (*P* = 0.006), the Gamma (*P* = 0.020) and the Delta (*P* = 0.029). In the solid organ transplants, the IC50 titers between the two vaccines were significantly different for the 2019-nCoV (*P* = 0.005), the Alpha (*P* = 0.005), the Gamma (*P* = 0.020), and Delta *P* = 0.029). In the autoimmune diseases, the IC50 titers between the two vaccines were significantly different for the 2019-nCoV (*P* = 0.017), the Alpha (*P* = 0.009), the Gamma (*P* = 0.020), and the Delta (*P* = 0.047).
At 3 months, in the healthy controls, the IC50 titers between the two vaccines were significantly different for the 2019-nCoV (*P* < 0.001) and all the other VOCs (*P* < 0.001). In the solid organ transplants, the IC50 titers between the two vaccines were significantly different for the 2019-nCoV (*P* = 0.005), the Alpha (*P* = 0.007), and the Gamma (*P* = 0.022). In the autoimmune diseases, the IC50 titers between the two vaccines were significantly different for the 2019-nCoV (*P* = 0.012), the Alpha (*P* = 0.035), the Beta (*P* = 0.036), the Gamma (*P* = 0.036), and the Delta (*P* = 0.035).
Resulting p-values were adjusted for multiple testing using the False Discovery Rate (FDR) approach of Benjamini-Hochberg.

© 2022 Obeid M et al. *JAMA Oncology.*

Supplemental Figure 5

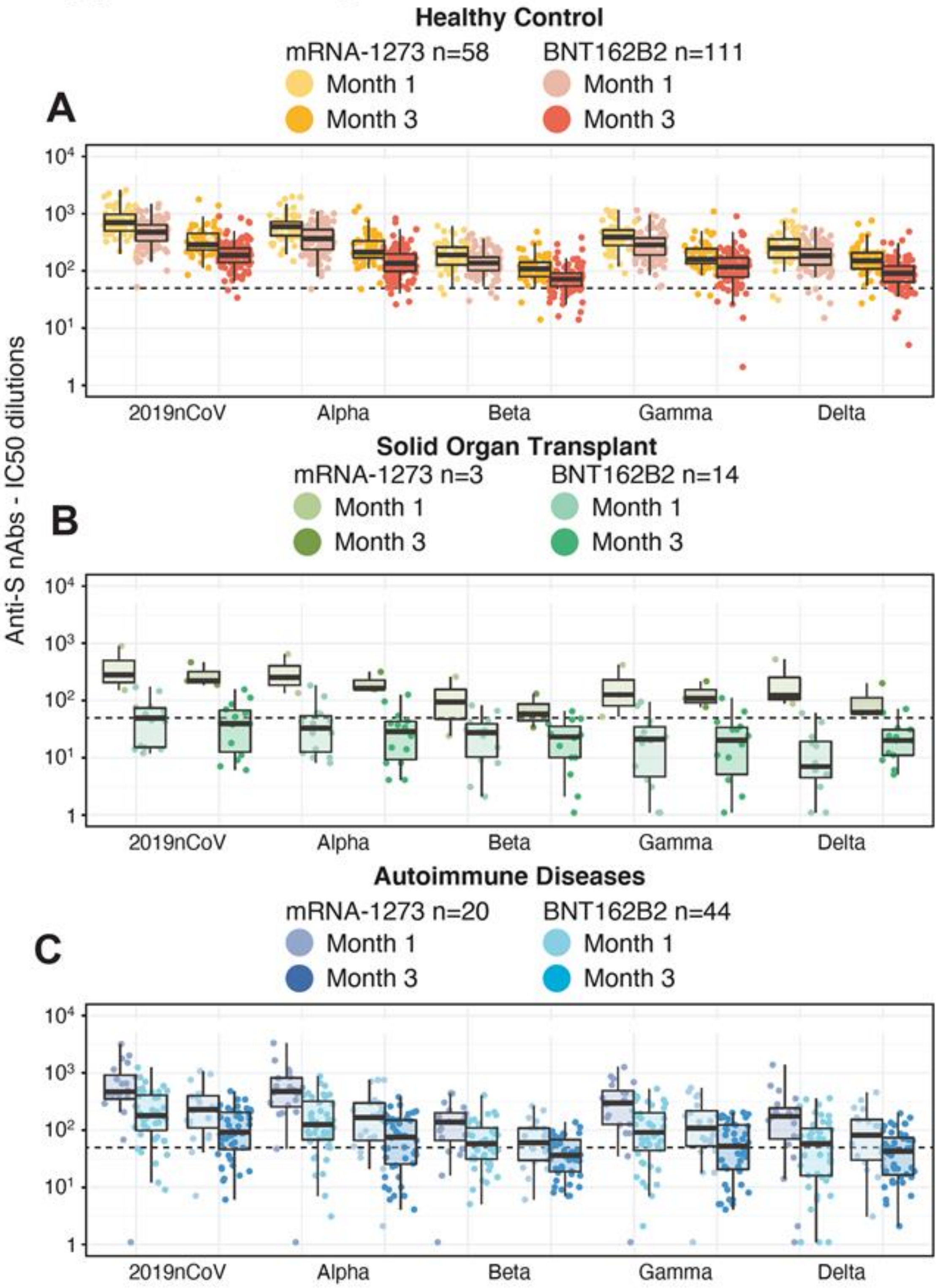

© 2022 Obeid M et al. *JAMA Oncology.*

eFigure 6. Proportion of participants with different levels of neutralizing antibody titers. IC50 titers were stratified as follows: <50 negative response; >50<100 weak response; >100<150 moderate response; <150 high response. The proportion of participants with different magnitude of IC50 titers was evaluated within each study population vaccinated with either the mRNA-1273 or the BNT162b2 vaccines.

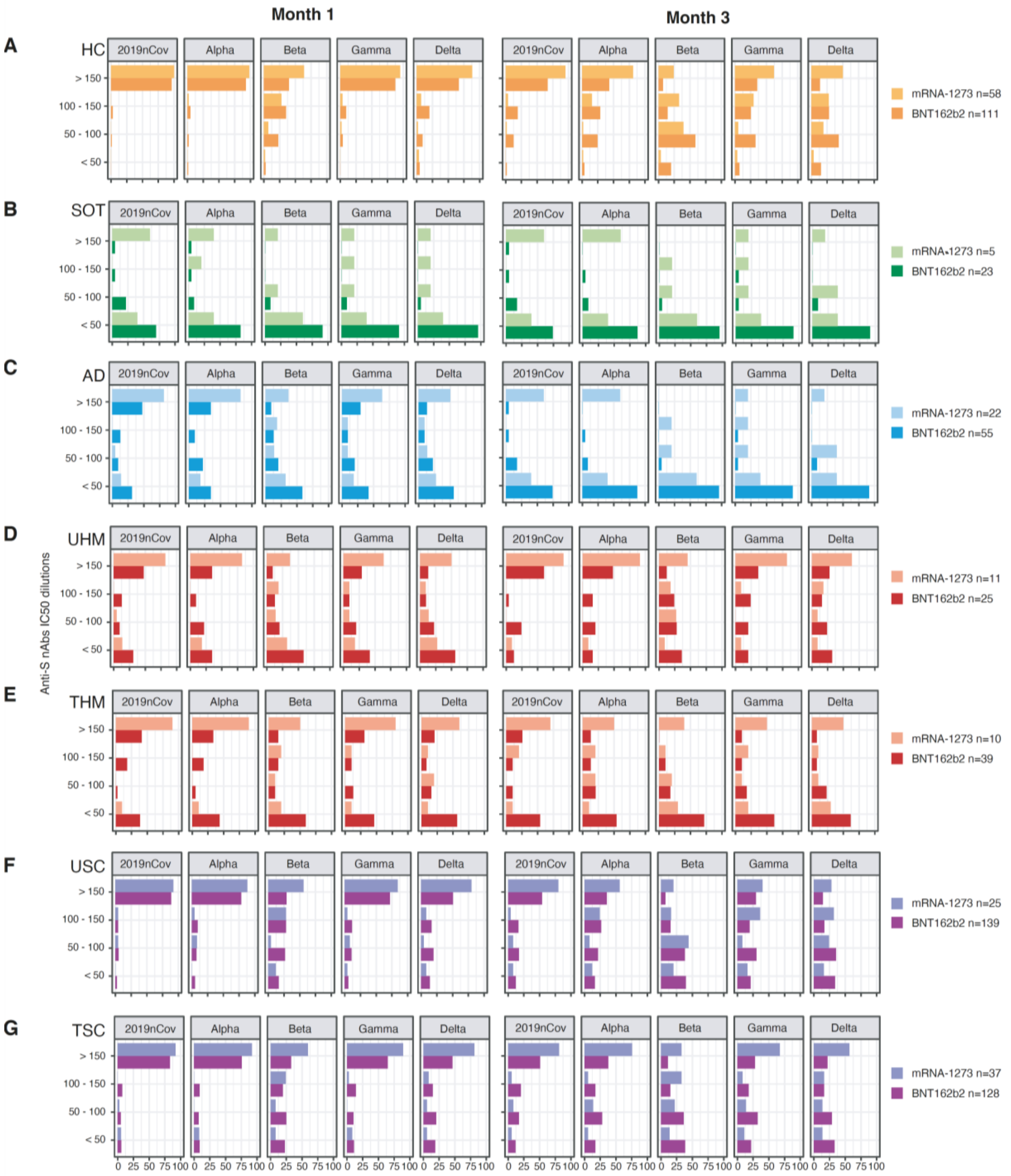

© 2022 Obeid M et al. *JAMA Oncology.*

eFigure 7. Proportion of participants with neutralizing antibodies responses at month 1, 3 and 6 after the 2nd Vaccination.
Healthy control (**A**), solid organ transplant (**B**) and autoimmune diseases (**C**) study populations are shown. Participants were combined for the analysis within each group.

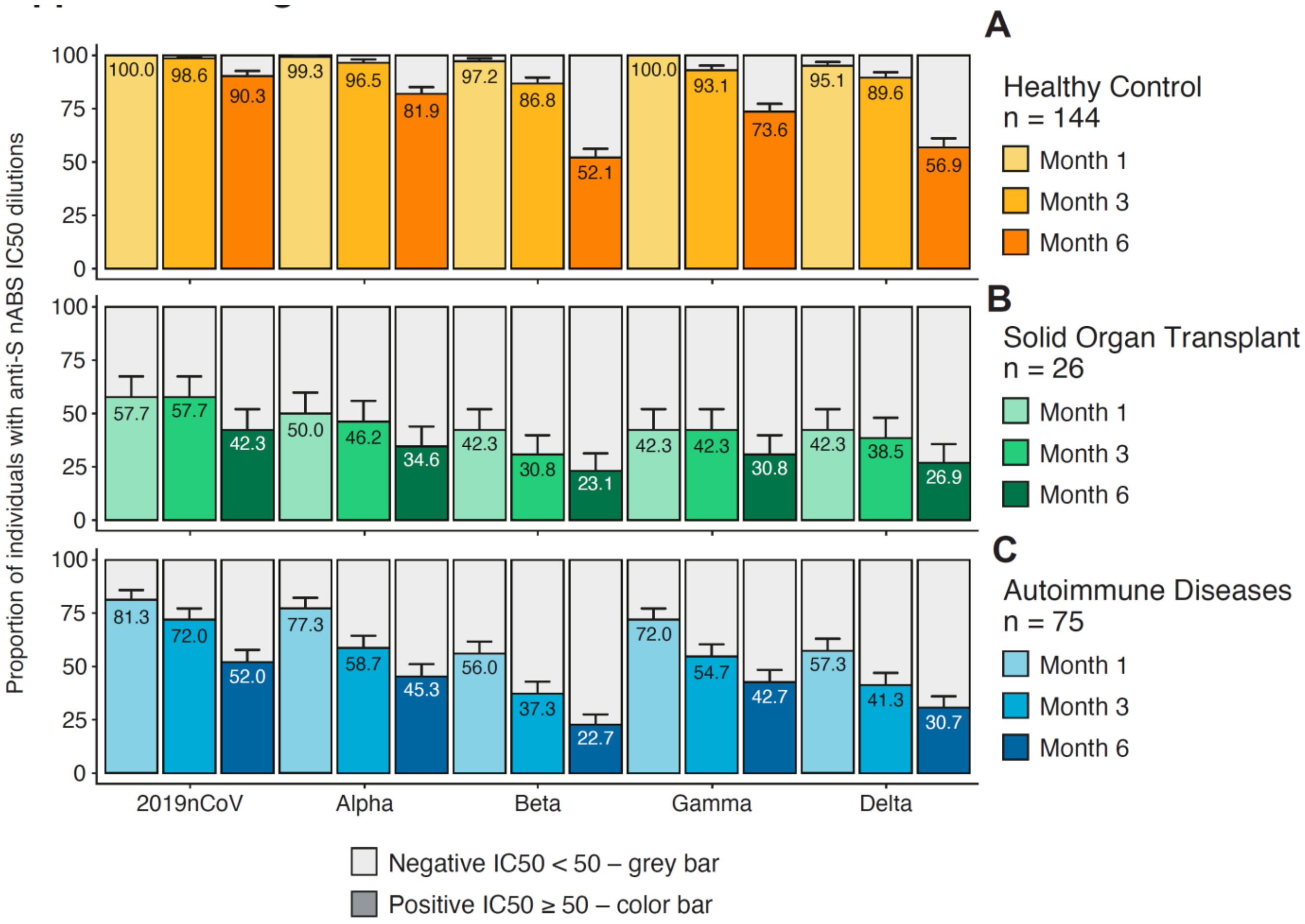

© 2022 Obeid M et al. *JAMA Oncology.*

eFigure 8. Estimates of the duration in time of binding response at month 6 ater the 2nd dose in the SC participants.

278 SC received BNT162b2 whereas 68 SC received mRNA-1273.The binding Abs duration in time (in weeks) was estimated by linear regression models using time as continuous covariate (the number of days corresponding to 1, 3 and 6 months after the second dose of vaccine).

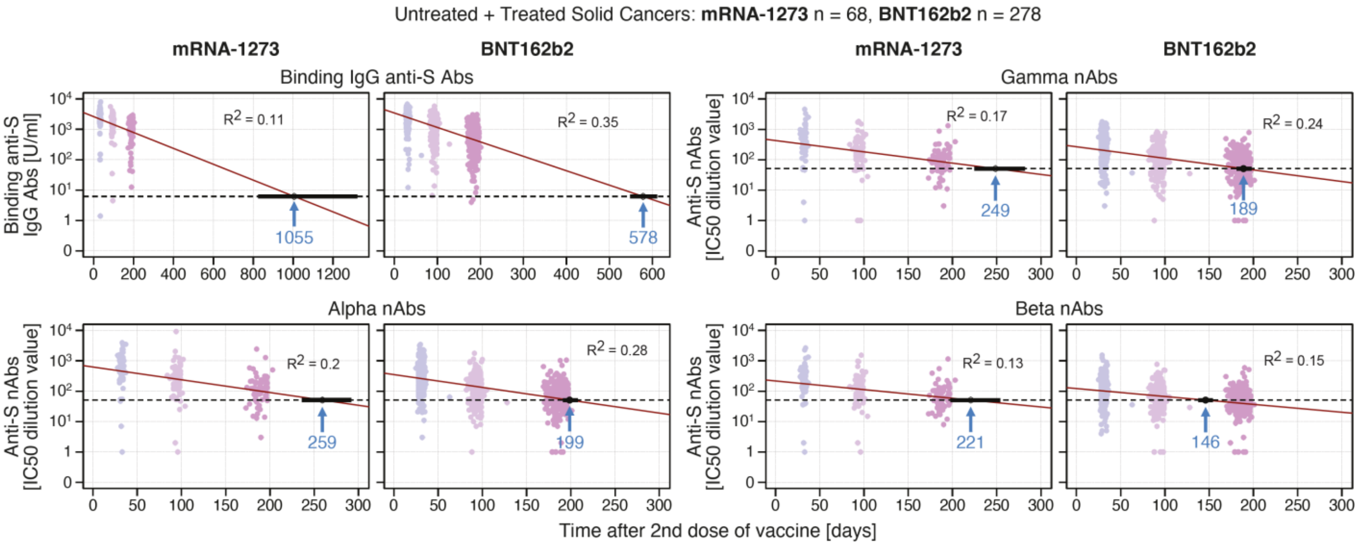

© 2022 Obeid M et al. *JAMA Oncology.*

eTable 5. Univariable linear regression models of binding and neutralizing antibodies at Month 1, 3 and 6 since the 2nd dose of vaccine

USC + TSC : **mRNA-1273** n = 68, **BNT162b2** n = 278

	mRNA-1273				BNT162b2			
	Coefficients	days	10%	90%	Coefficients	days	10%	90%
IgG (U/ml)		1005	821	1308		578	549	610
intercept	3.41822				3.53776			
slope	-0.00261				-0.00475			
n2019CoV		286	260	321		226	217	236
intercept	2.95560				2.69963			
slope	-0.00437				-0.00439			
Alpha		259	235	291		199	191	208
intercept	2.79578				2.54670			
slope	-0.00420				-0.00422			
Beta		221	198	253		146	138	154
intercept	2.33740				2.08685			
slope	-0.00285				-0.00260			
Gamma		249	225	282		189	181	198
intercept	2.63158				2.42755			
slope	-0.00372				-0.00381			
Delta		226	207	253		161	154	170
intercept	2.54210				2.19173			
slope	-0.00369				-0.00300			

HC: **mRNA-1273** n = 43, **BNT162b2** n = 101

	mRNA-1273				BNT162b2			
	Coefficients	days	10%	90%	Coefficients	days	10%	90%
IgG (U/ml)		1261	1081	1513		1061	945	1212
intercept	3.45941				3.36761			
slope	-0.00212				0.00243			
n2019CoV		290	271	311		252	239	267
intercept	2.95674				2.79981			
slope	-0.00431				-0.00433			
Alpha		250	236	268		216	206	226
intercept	2.85460				2.68890			
slope	-0.00458				-0.00455			
Beta		217	199	242		185	173	199
intercept	2.31535				2.22657			
slope	-0.00279				-0.00281			
Gamma		249	232	270		212	201	225
intercept	2.65257				2.55406			
slope	-0.00380				-0.00399			
Delta		233	216	255		186	176	197
intercept	2.48638				2.36550			
slope	-0.00334				-0.00354			

UHM + THM n = 79

	Coefficient	days	10%	90%
IgG (U/ml)		592	493	744
intercept	3.21765			
slope	-0.00410			
n2019CoV		208	184	243
intercept	2.53615			
slope	-0.00398			
Alpha		179	158	207
intercept	2.39801			
slope	-0.00387			
Beta		113	95	132
intercept	2.01459			
slope	-0.00272			
Gamma		160	140	189
intercept	2.23974			
slope	-0.00332			
Delta		127	105	154
intercept	2.01927			
slope	-0.00245			

Analysis performed on log10-transformed data (Binding-IgG (U/ml) and neutralization (IC50 dilution values))

Analysis performed on binding and neutralizing antibodies log10-transformed values.

© 2022 Obeid M et al. *JAMA Oncology.*

eFigure 9. Estimates of the duration in time of neutralizing response at month 6 since the 2nd dose in the HC participants.

101 HC received BNT162b2 whereas 43 HC received mRNA-1273. The neutralization Abs duration in time (in weeks) against the Alpha, Beta and Gamma VOC was estimated by linear regression models using time as continuous covariate (the number of days corresponding to 1, 3 and 6 months after the second dose of vaccine).

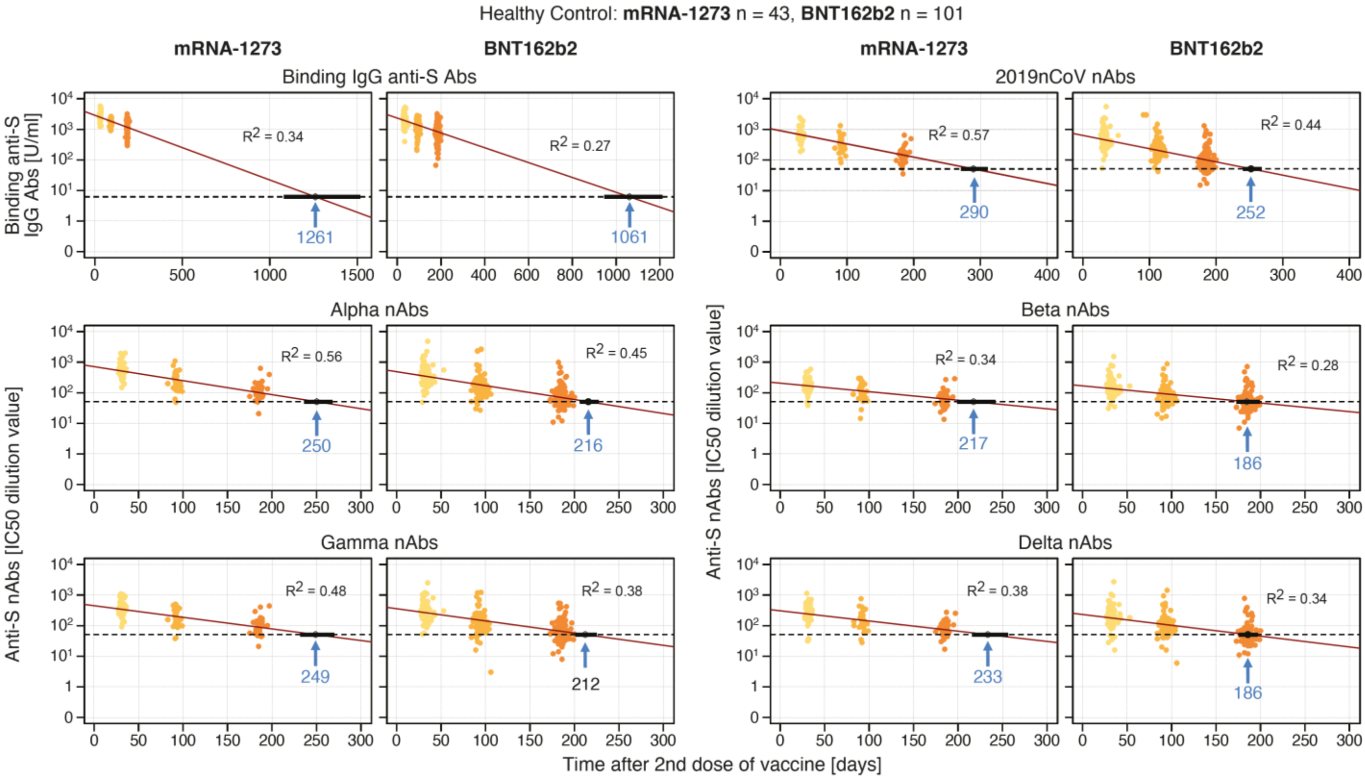

© 2022 Obeid M et al. *JAMA Oncology.*

eFigure 10. Estimates of the duration in time of neutralizing response at month 6 since the 2nd dose in the HM participants.

49 HM received BNT162b2 whereas 30 HM received mRNA-1273. The neutralization Abs duration in time (in weeks) against the Alpha, Beta and Gamma VOCs was estimated by linear regression models using time as continuous covariate (the number of days corresponding to 1, 3 and 6 months after the second dose of vaccine).

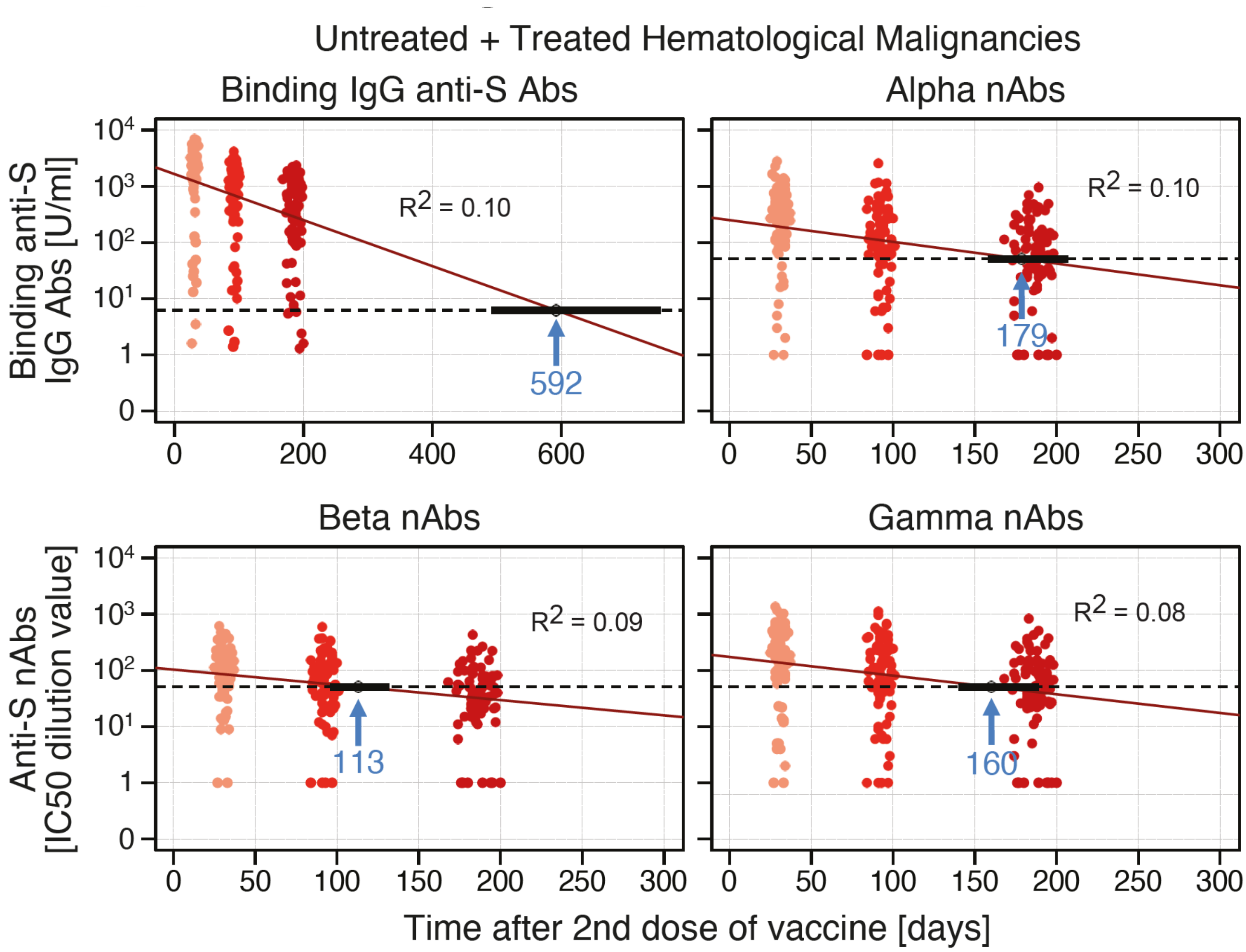

© 2022 Obeid M et al. *JAMA Oncology.*

eFigure 11. Percentage of participants reporting local and systemic Reactions Reported at V2 visit after Injection of BNT162b2 or mRNA-1273.

Data on local and systemic reactions were collected from 838 participants at visit V2, week 1 after the second vaccine. Solicited injection-site (local) reactions are shown in Panel A. Pain at the injection site was assessed according to the following scale: mild, does not interfere with activity; moderate, interferes with activity; severe, prevents daily activity; and grade 4, emergency department visit or hospitalization. Redness and swelling were measured according to the following scale: mild, 2.0 to 5.0 cm in diameter; moderate, >5.0 to 10.0 cm in diameter; severe, >10.0 cm in diameter; and grade 4, necrosis or exfoliative dermatitis (for redness) and necrosis (for swelling). Systemic events and medication use are shown in Panel B. Fever was assessed according to the following scale: mild; temperature 38.0 to 38.4°C, moderate; temperature >38.4 to 38.9°C severe; temperature >38.9 to 40.0°C, grade 4; temperature >40.0°C. Additional scales were as follows: fatigue, headache, chills, new or worsened muscle pain, new or worsened joint pain (mild: does not interfere with activity; moderate: some interference with activity; or severe: prevents daily activity), vomiting (mild: 1 to 2 times in 24 hours; moderate: >2 times in 24 hours; or severe: requires intravenous hydration), and diarrhea (mild: 2 to 3 loose stools in 24 hours; moderate: 4 to 5 loose stools in 24 hours; or severe: 6 or more loose stools in 24 hours); grade 4 for all events indicated an emergency department visit or hospitalization.

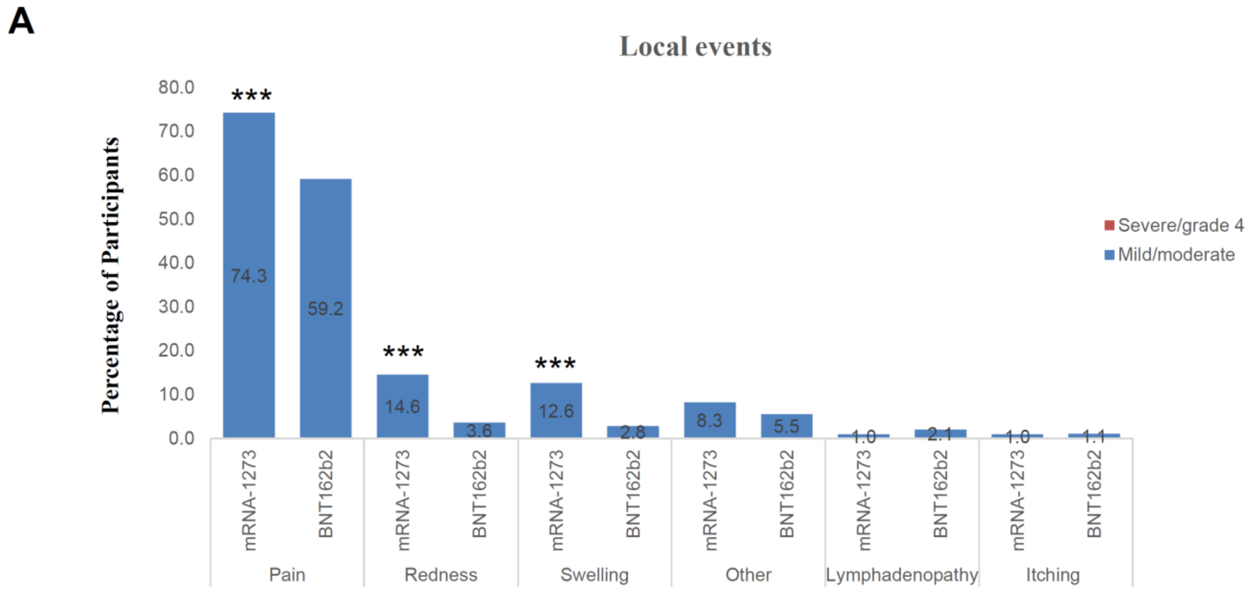

© 2022 Obeid M et al. *JAMA Oncology.*

B

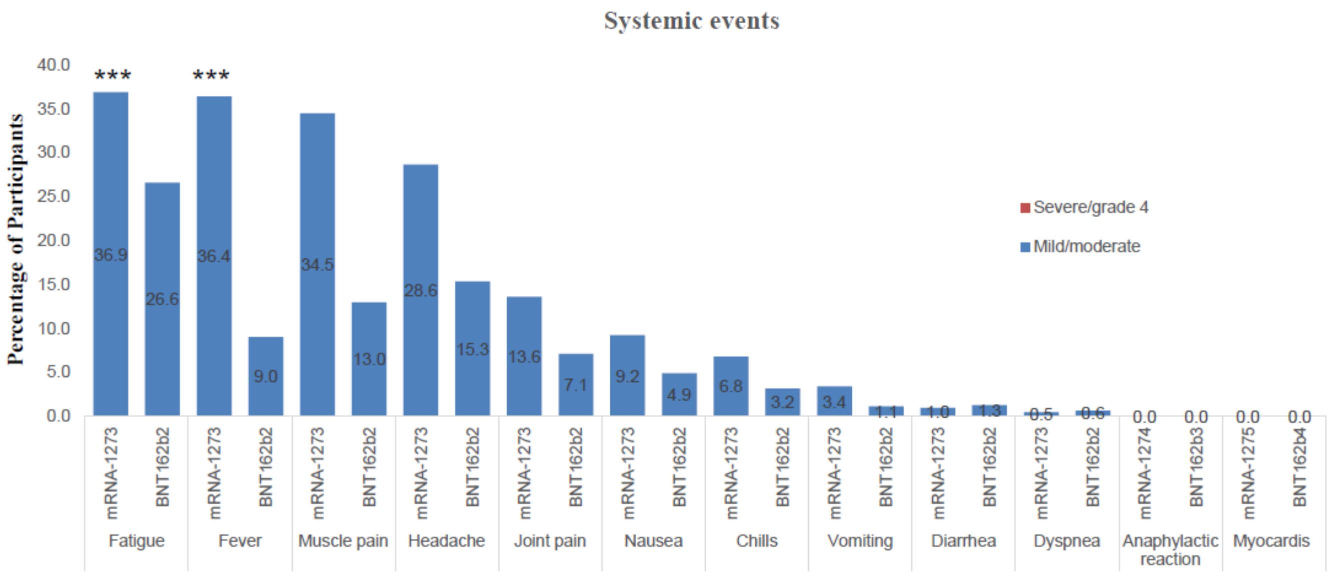

© 2022 Obeid M et al. *JAMA Oncology.*

eReferences

1. Fenwick C, Croxatto A, Coste AT, et al. Changes in SARS-CoV-2 Spike versus Nucleoprotein Antibody Responses Impact the Estimates of Infections in Population-Based Seroprevalence Studies. *J Virol*. Jan 13 2021;95(3)doi:10.1128/JVI.01828-20
2. Fenwick C, Turelli P, Pellaton C, et al. A high-throughput cell- and virus-free assay shows reduced neutralization of SARS-CoV-2 variants by COVID-19 convalescent plasma. *Sci Transl Med*. Aug 4 2021;13(605)doi:10.1126/scitranslmed.abi8452
3. Benjamini Y, Hochberg Y. Controlling the False discovery rate – a practical and powerful approach to multiple testing. *Journal of the Royal Statistical Society*. 1995;

© 2022 Obeid M et al. *JAMA Oncology.*
